# Quantitative Trait Loci Mappings for the Sulfur Utilization Efficiency-Related Traits at the Seedling Stage of Wheat

**DOI:** 10.3390/genes15121550

**Published:** 2024-11-29

**Authors:** Longteng Ma, Jiali Li, Hui Wang, Yunhui Zhai, Qing Xu, Hongling Yang, Yizheng Li, Ying Guo, Fanmei Kong, Sishen Li, Yan Zhao

**Affiliations:** 1 State Key Laboratory of Wheat Improvement, College of Agronomy, Shandong Agricultural University, Tai’an 271018, China; 19861909357@163.com (L.M.); 18864820750@163.com (H.W.); 15615744258@163.com (Y.Z.); xq2000824@163.com (Q.X.); 19105425886@163.com (H.Y.); liyzh1002@163.com (Y.L.); guoying@sdau.edu.cn (Y.G.); ssli@sdau.edu.cn (S.L.); 2 Tai’an Subcenter of National Wheat Improvement Center, College of Agronomy, Shandong Agricultural University, Tai’an 271018, China; 3 National Engineering Laboratory for Efficient Utilization of Soil and Fertilizer Resources, Shandong Agricultural University, Tai’an 271018, China; lijiali9129@163.com (J.L.); fmkong@sdau.edu.cn (F.K.); 4 School of Education Science, Jiangsu Second Normal University, Nanjing 210013, China

**Keywords:** common wheat, sulfur (S), utilization efficiency, recombinant inbred line (RIL), quantitative trait locus (QTL)

## Abstract

Background: Sulfur (S) is a vital element for the normal growth and development of plants, performing crucial biological functions in various life processes. Methods: This study investigated thirteen S utilization efficiency (SUE)-related traits at the seedling stage of wheat using a recombinant inbred line (RIL) population. The quantitative trait loci (QTLs) were mapped by genetic mapping. Thirteen S utilization efficiency-related traits were investigated under two hydroponic culture trials with low S (0.1S, T1), moderate S (0.5S, T2), and high S (1.5S, T3) levels, using the wheat RILs. Results: A total of 170 QTLs for the thirteen traits in different treatment environments were identified. Among them, 89, 103, and 101 QTLs were found in T1, T2, and T3, respectively. A total of 63 QTLs were found in the multiple treatment environments, the other 107 QTLs only being detected in a single treatment environment. Among them, thirteen relatively high-frequency QTLs (RHF-QTLs) and eleven QTL clusters were found. Five (*QSh-1D*, *QRn-1D*, *QSdw-1D*, *QTdw-1D*, and *QTsc-1D*) and six (*QRdw-6A*, *QSdw-6A*, *QTdw-6A*, *QRsc-6A*, *QSsc-6A*, and *QTsc-6A*) RHF-QTLs were identified in QTL clusters C3 and C10, respectively. Conclusion: These thirteen RHF-QTLs and eleven QTL clusters are expected to apply to the molecular marker-assisted selection (MAS) of wheat.

## 1. Introduction

Sulfur (S) is a nutritional element that is indispensable for plants, acknowledged as the fourth most important nutrient after nitrogen (N), phosphorus (P), and potassium (K) [1]. During plant growth, S plays a vital role in structural, catalytic, defensive, and metabolism processes. S promotes the synthesis of several amino acids, vitamins, coenzymes, and proteins, thereby promoting various physiological processes [2,3,4]. It is involved in the formation of sulfhydryl (S-H), disulfide bonds (S-S) [5], cysteine, methionine [6], and chlorophyll and promotes root growth and seed development [1]. It has been shown that S nutrition can relieve the negative effects of environmental stress [7,8,9,10]. However, the increased use of unbalanced fertilizers, primarily S-free ones, and the decreased discharge of industrial S into the atmosphere have led to widespread S deficiency in many agricultural systems, which has become an important constraint on crop production [11,12,13]. Insufficient supply of S could inhibit photosynthesis and protein synthesis [14] and decrease the osmotic potential of cell sap [15]. Thus, S deficiency affects yield and quality of the crop [16,17,18,19].

Wheat (*Triticum aestivum* L.) is a globally significant staple food crop. The yield and quality of wheat are greatly affected by soil nutrition. A variety of studies have demonstrated that S and N are intimately associated in plant metabolism [19,20,21] and that S can influence nitrogen utilization efficiency in plants [22,23,24]. Salvagiotti et al. [23] found that S fertilizer application improves the rate of N uptake prior to anthesis and thus increases the final grain yield. Without an adequate supply of S, wheat cannot use nitrogen efficiently [4]. The availability of S is crucial in the major storage proteins’ biosynthesis in wheat. N and S fertilizer at anthesis can increase the grain protein. It can also improve the stability, swelling, and extensibility of the dough, as well as improving the gluten network [25,26,27]. S fertilizers affect the composition of the protein and, mainly, raise the level of gluten [28,29]. According to prior study results, the utilization of S enhances the activities of nitrate reductase and glutamine synthetase in flag leaves, which in turn influences the amount of various protein components [30,31,32]. S availability has been shown to be positively related to gluten production and negatively related to the ratio of glutenin to glutenin [33]. Therefore, sufficient S fertilizer is essential to ensure optimum growth and yield of wheat in proper growth stages [34,35].

A quantitative trait locus (QTL) analysis is a common method to study the genetic loci of complex quantitative traits [36]. Nutrient utilization efficiency-related traits are suitable to be studied by this method. The research on the utilization efficiency of macroelements in wheat by QTL mainly focused on nitrogen, phosphorus, and potassium [37,38,39,40,41,42,43,44,45,46]; others were about grain mineral nutrient concentrations [47,48]. However, little is known about the S utilization efficiency in wheat.

The purpose of our study was to conduct a QTL analysis using a recombinant inbred line (RIL), to identify QTLs of the S utilization efficiency-related traits at the seedling stage in wheat.

## 2. Materials and Methods

### 2.1. Plant Materials

A total of 131 lines from the RIL population were used for the construction of the genetic map and the investigation of the phenotype. This RIL population was obtained by the single-seed descent (SSD) method using the hybrid combination “Chuan 35050 × Shannong 483” [49,50]. Chuan 35050 and Shannong 483 were the varieties planted in the southwestern and Huang-Huai winter wheat area of China, respectively. Among them, Shannong 483 was bred from “Ai-Meng-Niu”, the one as the backbone parent of wheat breeding in China.

### 2.2. Experimental Design

The experiment was carried out for two years at the Shandong Agricultural University experimental station. The 131 RIL populations and their parents were subjected to two independent hydroponic trials in a greenhouse, conducted in succession. Three S concentration treatments were designed: low S (0.1S, T1), moderate S (0.5S, T2), high S (1.5S, T3) (Table 1). Hoagland’s nutrient solution [51] was used with some amelioration to optimize wheat growth (Table S1). Each treatment was repeated three times. Three environments were designed: the first trial and the second trial were set as E1 and E2, respectively, and the average value (AV) of the same treatments in both trials was used as the third environment for the QTL analysis.

A total of 100 seeds were collected for each line and its parents. Prior to use, the seeds were sterilized with 10% H_2_O_2_ solution for five minutes. This was followed by a washing step with distilled water. The seeds were then germinated in Petri dishes for a period of seven days using moistened filter paper. For each replication, three uniformly growing seedlings were selected from each line. These seedlings were required to have both primary roots and germinal sheaths measuring between three and four centimeters in length. The selected seedlings underwent fixation with two sponges and were subsequently placed on a perforated tray. The trays were then placed in plastic buckets with twenty liters of a nutrient solution.

This nutrient solution was renewed weekly for 15 days and every 3 days for the remaining 25 days. Additionally, 0.1 mmol/L HCl and diluted NaOH were used to keep a PH range between 6.0 and 6.2 and keep ventilation. After being cultivated in the completely nutrient solution for 40 days, we washed the plants with the distilled water, which was absorbed with the absorbent paper. We put the seedling and root in an envelope, respectively, green-removing at 80 °C and stoving at 40 °C; weighed the dry weight; and screened the powder with nylon mesh.

### 2.3. Trait Measurement

A total of 13 seedling traits were investigated, including three morphological traits (SH, MRL, and RN), four biomass traits (RDW, SDW, TDW, and RSR), three S uptake efficiency (SUpE) traits (RSC, SSC, and TSC), and three S utilization efficiency (SUtE) traits (RSUE, SSUE, and TSUE) (Table 2). The plants should be harvested on the 40th day of the seedling stage. The roots should then be rinsed with distilled water for a minimum of ten minutes, after which the excess water should be removed using blotting paper. For each plant, SH, MRL, and RN were first counted and measured. The shoots and roots were then cut off with scissors. The moistened roots and seedlings were placed in an oven to be dried. The remaining traits were determined according to the methods listed in Table 2. To ensure ease of data investigation and accuracy, a mixture of experimental materials from the same S treatment was used to measure the traits.

### 2.4. Data Analysis

The SAS 9.3 software program (SAS Institute, Cary, NC, USA) was used to analyze the variance (ANOVA) and partial correlation coefficients among the treatments and investigated traits. The heritability (*h_B_*^2^) was calculated by SAS, *h_B_*^2^ = *σ_g_*^2^/(*σ_g_*^2^ + *σ_e_*^2^), where *σ_g_*^2^ is the genotypic variance, and *σ_e_*^2^ is the total error variance [52]. To infer statistical significance, we opted for Student’s *t*-test, and considered *p* < 0.05 as an acceptable level of statistical significance.

### 2.5. QTL Analysis

For the QTL analysis, an enriched genetic map [50] was used. The markers used in mapping are mostly DArTs (Diversity Array Technology), SSRs (Simple Sequence Repeats), EST-SSRs, and other molecular and biochemical loci. This map consists of 719 markers. They are distributed on 21 chromosomes. The total map length is 4008.4 cM and the marker density is 7.15 cM. QTL mapping was performed using Windows QTL Cartographer 2.5 [53] in this study. Composite interval mapping (CIM) was used to locate QTLs. We selected the “model 6 standard analysis” and controlled the walk speed of 1 cM. In order to take into account the genetic background, regression was chosen for the “forward and backward” marker selection. In order to exclude closely linked control markers at the tested site, the blocked window size was set at 10 cM, with a five-control-marker maximum. The declaration of a significant QTL was set at a *p*-value of 0.05 or less, with a minimal LOD value of 3.0. These parameters were defined using 1000 permutations [54]. Multiple (two and more) QTLs for a trait were defined as the same QTL if they were identified at the same location in different S treatment environments [40]. In instances where three or more quantitative trait loci exhibited coincident confidence intervals (CIs), we designated this as a QTL cluster (LOD ≥ 2.5). A QTL that was identified in more than three treatment environments was regarded as a relatively high-frequency QTL (RHF-QTL) [39,55].

## 3. Results

### 3.1. Phenotypic Variation and Correlations Between Traits

The RIL population exhibited a considerable degree of variation (Table S2). The coefficient of variation exhibited considerable variation, ranging from 7.95% for SH under the high-S environment to 28.37% for RSC under the low-S environment. The analysis of variance (ANOVA) showed that the variance of genotypic effects was significant at the *p* < 0.01 level for all 13 traits (Table S3). For the majority of the 13 trait–treatment combinations, there was evidence of transgressive segregation. Thirteen traits were observed to be continuously distributed in all treatment environment combinations, and the heritability was high, ranging from 54.99% to 77.33%.

The response of seedling growth and S utilization efficiency of the wheat RIL populations to different S concentrations indicated that all nine traits, except for RDW, RSR, and three S utilization efficiency traits (RSUE, SSUE, and TSUE), exhibited a tendency to increase with increasing sulfur concentration. It demonstrated that increasing the S concentration had a certain promotion effect on seedling growth of wheat but reduced the S utilization efficiency of the plants.

Most of the correlation coefficients between 13 traits were significant except for nine *r*-values (Table S4). The correlation between SH and RSR is negative while the correlation between SH and other traits is positive. MRL is positively correlated with other traits, except the NR, SSUE, and TSUE. A significant positive correlation was observed between RDW and other traits. SDW was significantly and positively correlated with other traits except RSR. RSR and SH were highly significantly negatively correlated at *p* ≤ 0.01. RSR and SSUE were significantly negatively correlated at *p* ≤ 0.05.

### 3.2. Major Characteristics of the QTLs

**Figure 1:**
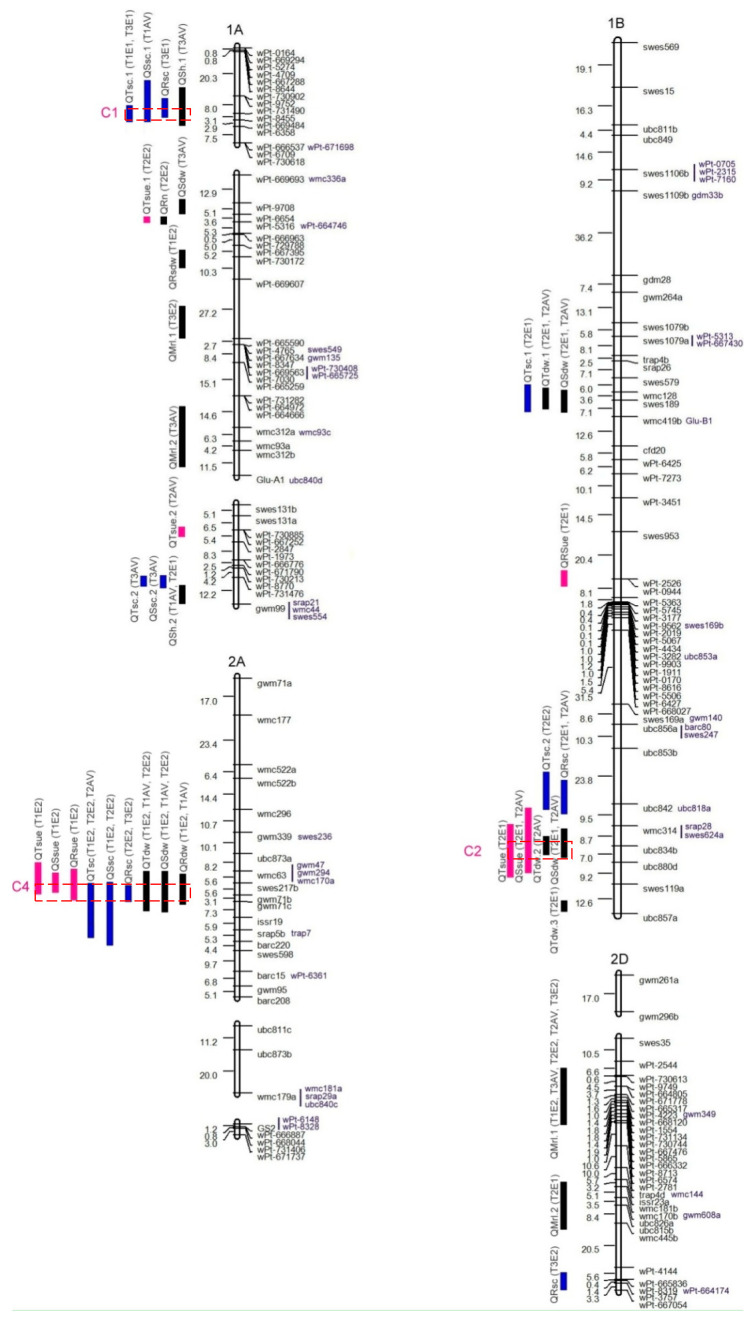

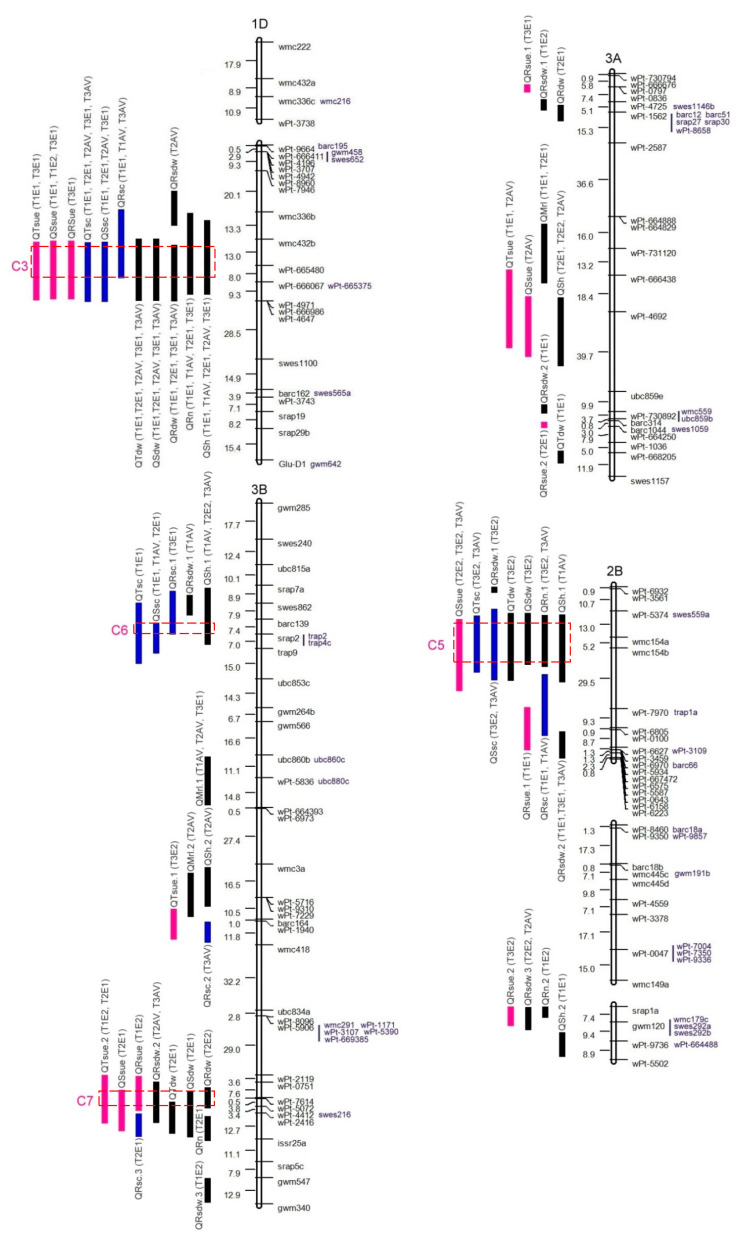

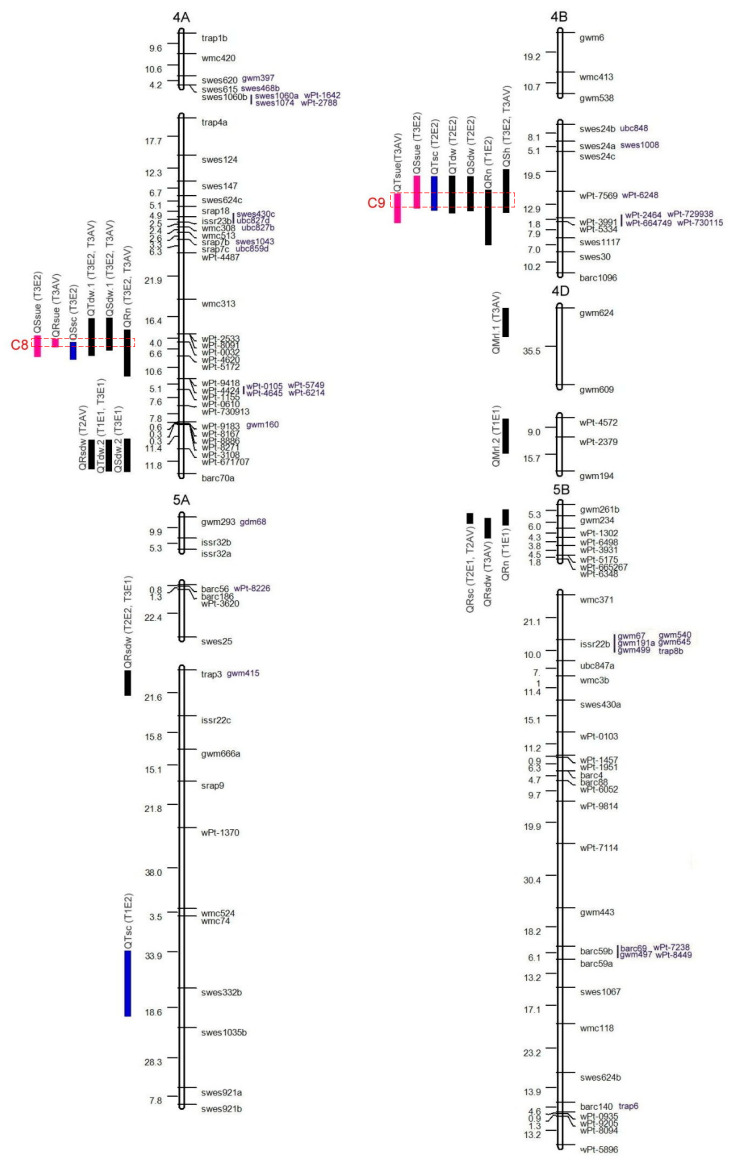

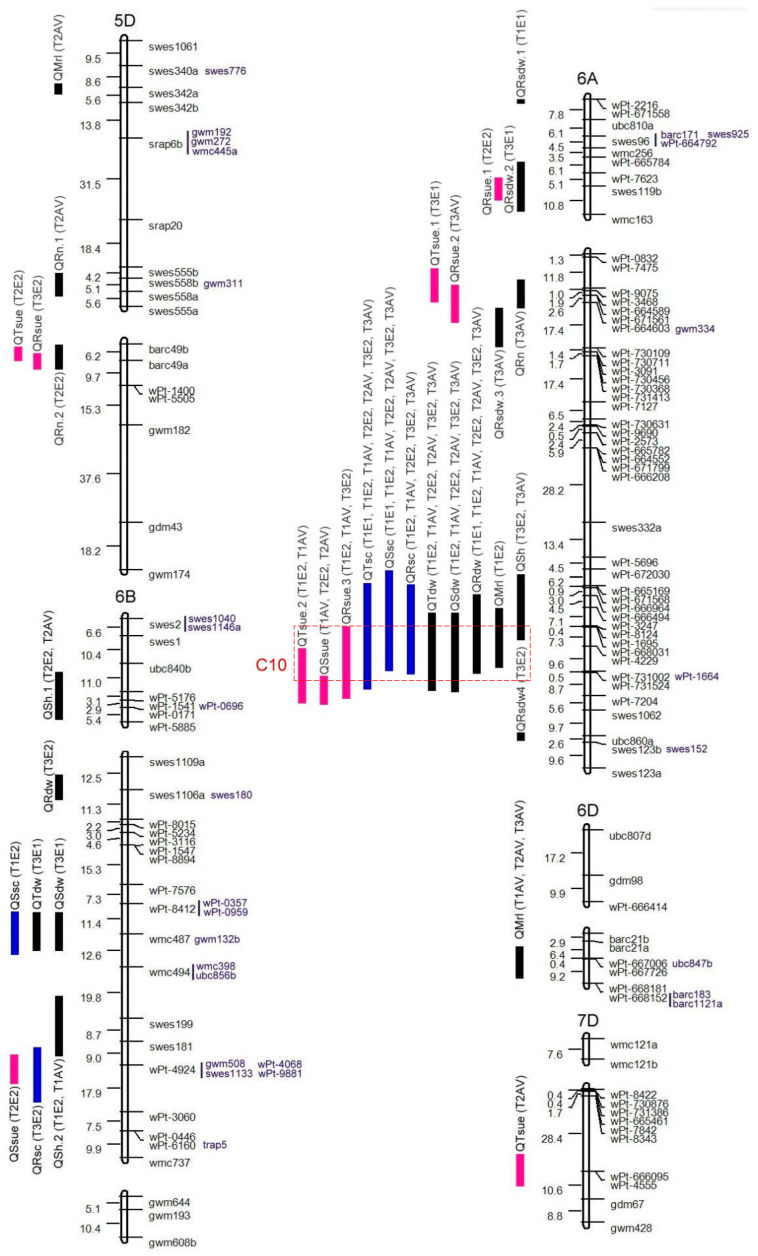

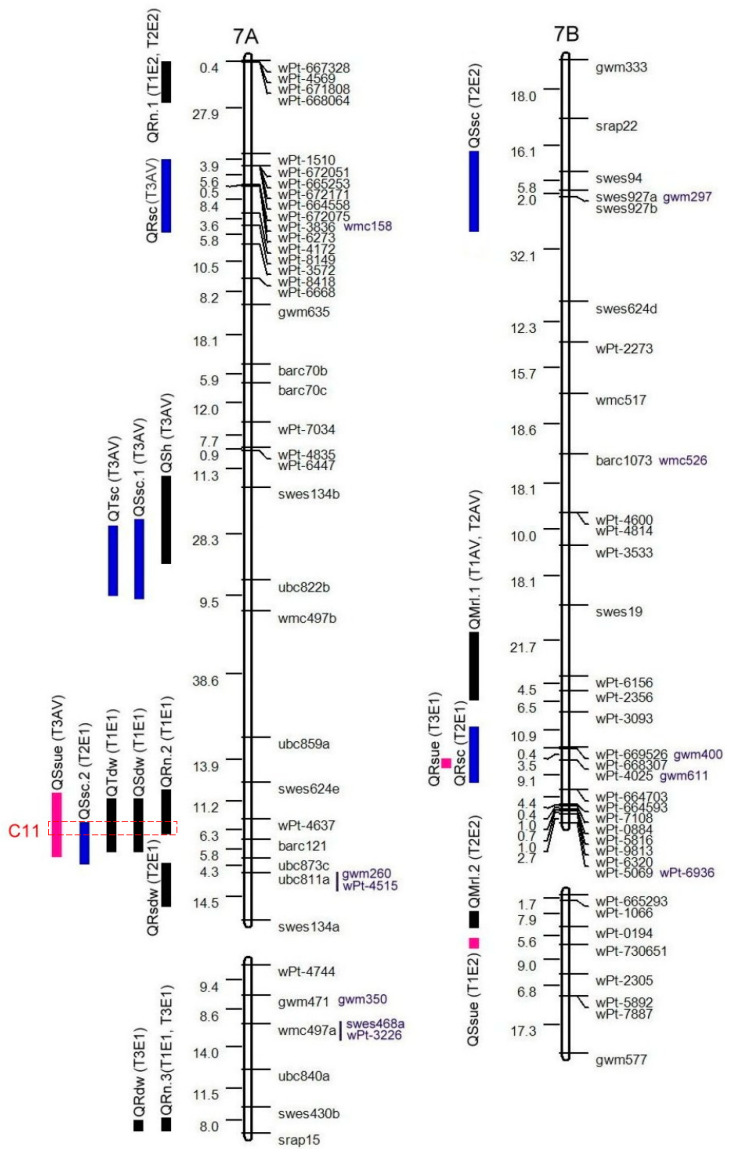
The chromosome distribution of QTLs in the RIL population. The colored bands on the left side of the chromosome indicate a QTL, and the marker names are on the right side. C1–C11 on the left side of the chromosome are indicated as 11 QTL clusters.

There were 170 QTLs detected on 21 chromosomes for 13 traits of the seedling (Figure 1). A total of 107 QTLs were detected only in a single treatment environment, while 63 QTLs were detected in multiple treatment environments. Of these, 41, 52, 38, and 39 QTLs were identified for the morphological traits (SH, MRL, and RN), the biomass traits (RDW, SDW, TDW, and RSR), the SUpE traits (RSC, SSC, and TSC), and the SUtE traits (RSUE, SSUE, and TSUE), respectively. A total of 78 QTLs exhibited a positive additive effect, indicating that the observed effect was enhanced by Chuan 35050. Conversely, 92 QTLs displayed a negative additive effect, suggesting that the effect was enhanced by Shannong 483. The phenotypic contributions of individual QTL ranged from 5.1% to 37.1% with a maximum LOD value of 10.3 (*QMrl.1-2D*, T2E2) (Table S5). Thirteen RHF-QTLs were found for ten traits (in addition to RSR, RSUE, and TSUE), with the average contributions ranging from 12.3% to 19.6% (Table 3). The research demonstrated that these RHF-QTLs were primarily situated on chromosomes 1D, 2D, and 6A. Of these, five RHF-QTLs (*QRdw-6A*, *QSdw-6A*, *QTdw-6A*, *QSsc-6A*, *QTsc-6A*) were detected in at least six treatment environments, thereby indicating that they are the more significant RHF-QTLs.

For SH, 13 QTLs were detected on ten chromosomes, viz. 1A, 1D, 2B, 2D, 3A, 3B, 4B, 6A, 6B, and 7A. The single QTL phenotypic contributions ranged from 7.8 to 26.5% in SH. One RHF-QTL (*QSh-1D*) was detected in five treatment environments; phenotypic contributions ranged from 10.9 to 26.5%. The average contribution of *QSh-1D* was 18.7%. The increasing effects of *QSh-1D* originated from Shannong 483.

For MRL, 14 QTLs were detected. They were distributed on nine chromosomes, 1A, 2D, 3A, 3B, 4D, 5D, 6A, 6D, and 7B. The single QTL phenotypic contributions ranged from 5.1 to 25.4% in MRL. One RHF-QTL (*QMrl.1-2D*) was detected in five treatment environments; phenotypic contributions ranged from 11.3 to 23.1%. The average contribution of *QMrl.1-2D* was 17.2%. The increasing effects of *QMrl.1-2D* originated from Shannong 483.

For RN, 14 QTLs were detected. They were distributed on ten chromosomes, 1A, 1D, 2B, 3B, 4A, 4B, 5B, 5D, 6A, and 7A. The single QTL phenotypic contributions ranged from 7.8 to 26.5% in RN. One RHF-QTL (*QRn-1D*) was detected in five treatment environments; phenotypic contributions ranged from 13.0 to 18.3%. The average contribution of *QRn-1D* was 15.7%. The increasing effects of *QRn-1D* originated from Shannong 483.

For RDW, seven QTLs were detected on seven chromosomes, viz. 1D, 2A, 3A, 3B, 6A, 6B, and 7A. An individual QTL explained 8.0–23.3% of the phenotypic variation in RDW. One RHF-QTL (*QRdw-6A*) was detected in seven treatment environments; phenotypic contributions ranged from 10.5 to 23.3%. The average contribution of *QRdw-6A* was 16.9%. The increasing effects of *QRdw-6A* originated from Chuan 35050.

For SDW, 13 QTLs were detected on 11 chromosomes, viz. 1A, 1B, 1D, 2A, 2B, 3B, 4A, 4B, 6A, 6B, and 7A. The single QTL phenotypic contributions ranged from 7.4 to 21.7% in SDW. Two RHF-QTLs, *QSdw-1D* and *QSdw-6A*, were detected in five and six treatment environments; phenotypic contributions ranged from 9.8 to 21.6% and 8.7 to 21.7%, respectively. The average contribution of *QSdw-1D* was 15.7% while that of *QSdw-6A* was 15.2%. The increasing effects of *QSdw-1D* and *QSdw-6A* originated from Shannong 483 and Chuan 35050, respectively.

For TDW, 14 QTLs were detected on 11 chromosomes, viz. 1B, 1D, 2A, 2B, 3A, 3B, 4A, 4B, 6A, 6B, and 7A. The single QTL phenotypic contributions ranged from 7.7 to 28.4% in TDW. Two RHF-QTLs, *QTdw-1D* and *QTdw-6A*, were detected in five and six treatment environments; phenotypic contributions ranged from 10.8 to 28.4% and 15.0 to 22.3%, respectively. The average contribution of *QTdw-1D* was 19.6% while that of *QTdw-6A* was 18.7%. The increasing effects of *QTdw-1D* and *QTdw-6A* originated from Shannong 483 and Chuan 35050, respectively.

For RSC, 14 QTLs were detected. They were distributed on 12 chromosomes, 1A, 1B, 1D, 2A, 2B, 2D, 3B, 5B, 6A, 6B, 7A, and 7B. An individual QTL explained 8.0–27.5% of the phenotypic variation in RSC. One RHF-QTL (*QRsc-6A*) was detected in five treatment environments; phenotypic contributions ranged from 10.4 to 27.5%. The average contribution of *QRsc-6A* was 19.0%. The increasing effects of *QRsc-6A* originated from Chuan 35050.

For SSC, 12 QTLs were detected on ten chromosomes, viz. 1A, 1D, 2A, 2B, 3B, 4A, 6A, 6B, 7A, and 7B. An individual QTL explained 6.4–20.7% of the phenotypic variation in SSC. One RHF-QTL (*QSsc-6A*) was detected in seven treatment environments; phenotypic contributions ranged from 7.8 to 17.1%. The average contribution of *QSsc-6A* was 12.5%. The increasing effects of *QSsc-6A* originated from Chuan 35050.

For TSC, 12 QTLs were detected on ten chromosomes, viz. 1A, 1B, 1D, 2A, 2B, 3B, 4B, 5A, 6A, and 7A. The single QTL phenotypic contributions ranged from 7.0 to 19.4% in TSC. Two RHF-QTLs, *QTsc-1D* and *QTsc-6A*, were detected in five and seven treatment environments; phenotypic contributions ranged from 10.3 to 15.5% and 9.6 to 19.4%, respectively. The average contribution of *QTsc-1D* was 12.9% while that of *QTsc-6A* was 14.5%. The increasing effects of *QTsc-1D* and *QTsc-6A* originated from Shannong 483 and Chuan 35050, respectively.

For SSUE, 12 QTLs were detected. They were distributed on 12 chromosomes, 1B, 1D, 2A, 2B, 3A, 3B, 4A, 4B, 6A, 6B, 7A, and 7B. The single QTL phenotypic contributions ranged from 7.2 to 37.1% in SSUE. One RHF-QTL (*QSsue-1D*) was detected in three treatment environments; phenotypic contributions ranged from 8.9 to 15.7%. The average contribution of *QSsue-1D* was 12.3%. The increasing effects of *QSsue-1D* originated from Chuan 35050.

### 3.3. QTL Clusters

There are 11 QTL clusters (C1-C11) that were found on chromosomes 1A, 1B, 1D, 2A, 2B, 3B, 4A, 4B, 6A, and 7A (Table 4). Two clusters, C3 and C10, were found to be associated with more than ten traits, indicating that the two loci were more important for the S utilization efficiency.

The cluster C3 on chromosome 1D had two, three, three, and three QTLs between marker intervals *wPt7946* and *GlluD1* for morphological traits, biomass weight, S content, and S utilization efficiency, respectively (Table 4, Figure 1). Among them, six RHF-QTLs (*QSh-1D*, *QRn-1D*, *QSdw-1D*, *QTdw-1D*, *QTsc-1D*, and *QSsue-1D*) were found. Except for *QSsue-1D*, the remaining five QTLs had negative additive effects, indicating that they were enhanced by Shannong 483 (Table S5). The cluster C10 on chromosome 6A had two, three, three, and three QTLs between marker region *wPt672030* and *swes1062* for the morphological traits, biomass weight, S content of seedlings, and S utilization efficiency, respectively (Table 4, Figure 1). Six RHF-QTLs (*QRdw-6A*, *QSdw-6A*, *QTdw-6A*, *QRsc-6A*, *QSsc-6A*, and *QTsc-6A*) were found among them. The additive effect was positive for all QTLs, indicating that they were enhanced by Chuan 35050 (Table S5).

## 4. Discussion

### 4.1. Impact of S Treatment on Seedling Traits in RILs

The lack of S in the soil results in the limitation of S content in plant tissues and limits the production of dry matter [11,12,56]. According to this study, different S treatments had a significant effect on the phenotypic variance of most of the investigated traits (Table S2). Under low-sulfur treatment, most of the seedling traits (SH, MRL, SDW, TDW, RSR, RSC, SSC, and TSC) decreased, while RDW, RSUE, SSUE, and TSUE increased. Similar results were obtained in the research of Kong et al. [40], using the same RIL population under low-potassium treatment.

In this study, all the *r*-values between morphological/biomass traits (SH, RN, RDW, SDW, and TDW) and SUE traits (RSC, SSC, TSC, RSUE, SSUE, and TSUE) were significantly positive, indicating that with increased biomass, the seedling SUpE and SUtE were higher. These findings are consistent with those reported by Sharma et al. [57] for soybean S utilization efficiency. Consequently, the morphological/biomass traits (SH, RN, RDW, SDW, and TDW) could be taken as the main morphological indicators to evaluate SUE at the seedling stage, avoiding the need to test all germplasm for S content, thus enhancing the efficiency of the screening process for wheat germplasm with high sulfur efficiency.

### 4.2. QTL Location and QTL Clusters

Most nutrient QTLs have been detected, including those associated with P in hydroponic trials [39,49,58], N in hydroponic trials [59,60,61] and field trials [62], and K in hydroponic trials [40,42]. There are very few reports of S utilization efficiency QTL. Previous studies have detected the location of QTL associated with S content through the analysis of genetic mechanisms regulating mineral nutrient concentration in wheat grain [47]. In this study, 170 QTLs for thirteen seedling traits were identified under three S concentration treatments. QTLs associated with nutrient use efficiency for N, P, and K were detected using the same RIL population [39,40,41].

A relatively stable QTL can be detected in multiple environments, which is significant for wheat breeding [63]. The present study identified 13 RHF-QTLs (*QSh-1D*, *QMrl-2D*, *QRn-1D*, *QRdw-6A*, *QSdw-1D,6A*, *QTdw-1D,6A*, *QRsc-6A*, *QSsc-6A*, *QTsc-1D,6A*, *QSsue-1D*), which were present in multiple treatment environments and demonstrated stability as QTLs, contributing up to 16% to the variation in phenotypic variance on average. In addition, *QSdw*, *QTdw*, and *QTsc* were on chromosomes 1D and 6A with opposite additive effects, respectively. This suggests that QTL controlling the same trait may have different responses on different chromosomes. This study identified a total of eleven QTL clusters, containing at least four traits. Both C3 and C10 were detected in seven treatments and RHF-QTLs were found, indicating that these clusters were relatively stable. Surprisingly, cluster C2 and C8 were only found in T2 and T3, respectively, suggesting that these QTLs respond to specific S levels. The growth of plant seedlings is significantly influenced by the availability of nutrients [64,65]. Zhang et al. [58] identified two QTL clusters, both of which were related to N utilization efficiency and agronomic traits. In our research, nine QTL clusters were also found, containing QTLs of morphological traits, biomass traits, and S utilization efficiency traits. Correlation coefficients between these traits were almost significantly positive. This indicates that S use efficiency and the agronomic traits could be improved simultaneously.

Guo et al. [39] found that N, P, and K exhibit a cooperative uptake and utilization relationship at both the QTL and the phenotypic levels. Compared with our QTL mapping results, some QTL locations had been detected in adjacent marker regions by previous studies (Table S6). For instance, the traits of S utilization efficiency, S content, and dry weight that are near the markers *wPt7946-GlluD1* on 1D coordinated with nitrogen, phosphorus, and potassium utilization efficiency, content, and dry weight in Guo et al.’s study [39]; potassium utilization efficiency, and content in Kong et al.’s study [40] and dry weight in Sun et al.’s study [41].

## Figures and Tables

**Table 1:** Summary of S treatments for the hydroponic culture.

Trials		Treatments		
		Name	Code	S Concentration
Hydroponic culture trial	E1	LS (T1)	T1E1	0.1 mmol·L^−1^
		MS (T2)	T2E1	0.5 mmol·L^−1^
		HS (T3)	T3E1	1.5 mmol·L^−1^
	E2	LS (T1)	T1E2	0.1 mmol·L^−1^
		MS (T2)	T2E2	0.5 mmol·L^−1^
		HS (T3)	T3E2	1.5 mmol·L^−1^

LS: low S; MS: moderate S; HS: high S.

**Table 2:** Summary of investigated traits and their measurement methods under hydroponic culture trial.

Abbreviation	Traits	Units	Methods of Trait Measurement
SH	shoot height per plant	cm	Measured with a ruler
MRL	maximum root length per plant	cm	Measured with a ruler
RN	root number per plant	number	Average number of nine plants
RDW	root dry weight per plant	mg∙plant^−1^	Oven-dried and weighed on 1/10,000 balances
SDW	shoot dry weight per plant	mg∙plant^−1^	Oven dried and weighed on 1/10,000 balances
TDW	total dry weight per plant	mg∙plant^−1^	RDW + SDW
RSR	root–shoot ratio	-	RDW/SDW
RSC	root sulfur content per plant	mg∙plant^−1^	Using a sequential plasma spectrometer (ICPS-7500, Japan)
SSC	shoot sulfur content per plant	mg∙plant^−1^	Using a sequential plasma spectrometer (ICPS-7500, Japan)
TSC	total sulfur content per plant	mg∙plant^−1^	RSC + SSC
RSUE	root sulfur utilization efficiency	mg^2^RDW∙μg^−1^RSC	RDW^2^/(RSC × 1000)
SSUE	shoot sulfur utilization efficiency	mg^2^SDW∙μg^−1^SSC	SDW^2^/(SSC × 1000)
TSUE	total sulfur utilization efficiency	mg^2^TDW∙μg^−1^TSC	TDW^2^/(TSC × 1000)

**Table 3:** Relative high-frequency QTLs (RHF-QTLs) detected in at least three treatment environments in hydroponic culture trial.

Traits	QTL	Treatments	Marker Intervals	Additive Effects			*R*^2^ (%)		
				Min	Max	Average	Min	Max	Average
SH	*QSh-1D*	T1E1,T1AV,T2E1,T2AV,T3E1	*wmc432b-swes1100*	−2.14	−0.97	−1.56	10.90	26.50	18.70
MRL	*QMrl.1-2D*	T1E2,T3AV,T2E2,T2AV,T3E2	*wPt2544-trap4d*	−1.82	−1.02	−1.42	11.30	23.10	17.20
RN	*QRn-1D*	T1E1,T1AV,T2E1,T2AV,T3E1	*wmc336b-wPt666067*	−0.72	−0.40	−0.56	13.00	18.30	15.65
RDW	*QRdw-6A*	T1E1,T1E2,T1AV,T2E2,T2AV,T3E2,T3AV	*wPt672030-wPt7204*	2.86	5.88	4.37	10.5	23.30	16.90
SDW	*QSdw-1D*	T1E1,T2E1,T2AV,T3E1,T3AV	*wmc432b-wPt4647*	−20.52	−9.62	−15.07	9.80	21.60	15.70
	*QSdw-6A*	T1E2,T1AV,T2E2,T2AV,T3E3,T3AV	*wPt668031-swes1062*	8.66	22.48	15.57	8.70	21.70	15.20
TDW	*QTdw-1D*	T1E1,T2E1,T2AV,T3E1,T3AV	*wmc432b-wPt665480*	−24.55	−11.95	−18.25	10.80	28.40	19.60
	*QTdw-6A*	T1E2,T1AV,T2E2,T2AV,T3E3,T3AV	*wPt3247-swes1062*	13.85	27.54	20.70	15.00	22.30	18.65
RSC	*QRsc-6A*	T1E2,T1AV,T2E2,T3E2,T3AV	*wPt672030-wPt731002*	0.01	0.02	0.02	10.40	27.50	18.95
SSC	*QSsc-6A*	T1E1,T1E2,T1AV,T2E2,T2AV,T3E2,T3AV	*wPt672030-wPt4229*	0.03	0.05	0.04	7.80	17.10	12.45
TSC	*QTsc-1D*	T1E1,T2E1,T2AV,T3E1,T3AV	*wmc432b-wPt665480*	−0.05	−0.04	−0.05	10.3	15.50	12.90
	*QTsc-6A*	T1E1,T1E2,T1AV,T2E2,T2AV,T3E2,T3AV	*wPt672030-wPt7204*	0.04	0.06	0.05	9.60	19.40	14.50
SSUE	*QSsue-1D*	T1E1,T3E1,T1E2	*wmc432b-GlluD1*	−6.25	6.52	0.14	8.90	15.70	12.30

**Table 4:** Clusters comprising QTLs for at least four traits.

Cluster Code	Chromosome	Marker Intervals	QTL Number	QTLs for Seedling Traits				Treatments
C1	1A	*wPt731490-wPt669484*	4	*QRsc-1A*	*QSh.1-1A*	*QSsc.1-1A*	*QTsc.1-1A*	T1E1,T3E1,T1AV,T3AV,
C2	1B	*wmc314-ubc880d*	4	*QSdw.2-1B*	*QSsue-1B*	*QTdw.2-1B*	*QTsue-1B*	T2E1,T2AV
C3	1D	*wPt7946-GlluD1*	11	*QRdw-1D*	*QRsc-1D*	*QSdw-1D*	*QSh-1D*	T1E1,T1AV,T1E2,T2E1,
				*QSsue-1D*	*QTdw-1D*	*QTsue-1D*	*QTsc-1D*	T2AV,T3E1,T3AV
				*QRn-1D*	*QRSue-1D*	*QSsc-1D*		
C4	2A	*swes217b-barc15*	9	*QRdw-2A*	*QR* *sue-2A*	*QSsue-2A*	*QTsue-2A*	T1E2,T1AV,T2E2,T2AV,
				*QRsc-2A*	*QSdw-2A*	*QSsc-2A*	*QTdw-2A*	T3E2
				*QTsc-2A*				
C5	2B	*wPt5374-wPt7970*	7	*QSdw-2B*	*QSsc-2B*	*QSsue-2B*	*QTdw-2B*	T1AV,T2E2,T3E2,T3AV
				*QTsc-2B*	*QRn.1-2B*	*QSh.1-2B*		
C6	3B	*swes862-ubc853c*	5	*QRs* *r.1-3B*	*QSh.1-3B*	*QRsc.1-3B*	*QSsc-3B*	T1E1,T1AV,T2E1,T2E2,
				*QTsc-3B*				T3E1,T3AV
C7	3B	*wPt0751-issr25a*	9	*QRs* *r.2-3B*	*QR* *sue-3B*	*QTsue.2-3B*	*QRdw-3B*	T1E2,T2E1,T2E2,T2AV,
				*QRn-3B*	*QRsc.3-3B*	*QSdw-3B*	*QSsue-3B*	T3AV
				*QTdw-3B*				
C8	4A	*wPt0032-wPt5172*	6	*QSdw.1-4A*	*QTdw.1-4A*	*QRn-4A*	*QR* *sue-4A*	T3E2,T3E2,T3AV
				*QSsc-4A*	*QSsue-4A*			
C9	4B	*swes24c-wPt5334*	7	*QSh-4B*	*QSdw-4B*	*QSsue-4B*	*QTdw-4B*	T1E2,T2E2,T3E2,T3AV
				*QTsc-4B*	*QTsue-4B*	*QRn-4B*		
C10	6A	*wPt672030-swes1062*	11	*QSsc-6A*	*QRsc-6A*	*QSh-6A*	*QMrl-6A*	T1E1,T1E2,T1AV,T2E2,
				*QR* *sue.3-6A*	*QSdw-6A*	*QTdw-6A*	*QRdw-6A*	T2AV,T3E2,T3AV
				*QSsue-6A*	*QTsc-6A*	*QTsue.2-6A*		
C11	7A	*wPt4637-barc121*	5	*QSsue-7A*	*QSsc.2-7A*	*QTdw-7A*	*QSdw-7A*	T1E1,T2E1,T3AV
				*QRn.2-7A*				

## Data Availability

All datasets generated for this study are included in the article/Supplementary Material; further inquiries can be directed to the first author.

## Supplementary Materials

The following supporting information can be downloaded at: https://www.mdpi.com/article/10.3390/genes15121550/s1, Table S1: Nutrient solution ingredients for wheat seedling growth; Table S2: Phenotypic analysis of seedling traits for RIL population under different S treatments; Table S3: ANOVA for 13 S utilization efficiency-related traits; Table S4: Correlation coefficient of seedling traits for RIL population; Table S5: Additive QTLs for the investigated traits in the three environments and their mean values (MVs); Table S6: Locations of QTL clusters of S-related traits in this paper and the other traits in previous studies.

## References

[1] Marschner H., Marschner H. (1995). Introduction, Definition, and Classificatin of Mineral Nutrients. Mineral Nutrition of Higher Plants.

[2] Leustek T., Martin M.N., Bick J.-A., Davies J.P. (2000). Pathways and Regulation of Sulfur Metabolism Revealed through Molecular and Genetic Studies. Annu. Rev. Plant Biol..

[3] Zamboni A., Celletti S., Zenoni S., Astolfi S., Varanini Z. (2017). Root Physiological and Transcriptional Response to Single and Combined S and Fe Deficiency in Durum Wheat. Environ. Exp. Bot..

[4] Yu Z., Juhasz A., Islam S., Diepeveen D., Zhang J., Wang P., Ma W. (2018). Impact of Mid-Season Sulphur Deficiency on Wheat Nitrogen Metabolism and Biosynthesis of Grain Protein. Sci. Rep..

[5] Saito K. (2000). Regulation of Sulfate Transport and Synthesis of Sulfur-Containing Amino Acids. Curr. Opin. Plant Biol..

[6] Koprivova A., Giovannetti M., Baraniecka P., Lee B.-R., Grondin C., Loudet O., Kopriva S. (2013). Natural Variation in the ATPS1 Isoform of ATP Sulfurylase Contributes to the Control of Sulfate Levels in Arabidopsis. Plant Physiol..

[7] Khan N.A., Anjum N.A., Nazar R., Iqbal N. (2009). Increased Activity of ATP-Sulfurylase and Increased Contents of Cysteine and Glutathione Reduce High Cadmium-Induced Oxidative Stress in Mustard Cultivar with High Photosynthetic Potential. Russ. J. Plant Physiol..

[8] Astolfi S., Zuchi S. (2013). Adequate S Supply Protects Barley Plants from Adverse Effects of Salinity Stress by Increasing Thiol Contents. Acta Physiol. Plant..

[9] Fatma M., Asgher M., Masood A., Khan N.A. (2014). Excess Sulfur Supplementation Improves Photosynthesis and Growth in Mustard under Salt Stress through Increased Production of Glutathione. Environ. Exp. Bot..

[10] Cao Y., Ma C., Yu H., Tan Q., Dhankher O.P., White J.C., Xing B. (2023). The Role of Sulfur Nutrition in Plant Response to Metal(Loid) Stress: Facilitating Biofortification and Phytoremediation. J. Hazard. Mater..

[11] Zhao F., Hawkesford M., McGrath S. (1999). Sulphur Assimilation and Effects on Yield and Quality of Wheat. J. Cereal Sci..

[12] De Ruiter J.M., Martin R.J. (2001). Management of Nitrogen and Sulphur Fertiliser for Improved Bread Wheat (Triticum Aestivum) Quality. N. Z. J. Crop Hortic. Sci..

[13] Bouranis D.L., Malagoli M., Avice J.-C., Bloem E. (2020). Advances in Plant Sulfur Research. Plants.

[14] Lee B.-R., Zaman R., Avice J.-C., Ourry A., Kim T.-H. (2016). Sulfur Use Efficiency Is a Significant Determinant of Drought Stress Tolerance in Relation to Photosynthetic Activity in *Brassica napus* Cultivars. Front. Plant Sci..

[15] Kusaka M., Ohta M., Fujimura T. (2005). Contribution of Inorganic Components to Osmotic Adjustment and Leaf Folding for Drought Tolerance in Pearl Millet. Physiol. Plant..

[16] Scherer H.W. (2001). Sulphur in Crop Production—Invited Paper. Eur. J. Agron..

[17] Ali I., Aydin G., Mehmet A., Sait A.M., Süleyman T., Figen E. (2003). Diagnosis of Sulfur Deficiency and Effects of Sulfur on Yield and Yield Components of Wheat Grown in Central Anatolia, Turkey. J. Plant Nutr..

[18] Singh S.P., Singh M.P. (2014). Effect of Sulphur Fertilization on Sulphur Balance in Soil and Productivity of Wheat in a Wheat–Rice Cropping System. Agric. Res..

[19] Castellari M.P., Poffenbarger H.J., Van Sanford D.A. (2023). Sulfur Fertilization Effects on Protein Concentration and Yield of Wheat: A Meta-Analysis. Field Crops Res..

[20] Hesse H., Nikiforova V., Gakière B., Hoefgen R. (2004). Molecular Analysis and Control of Cysteine Biosynthesis: Integration of Nitrogen and Sulphur Metabolism. J. Exp. Bot..

[21] Dai Z., Plessis A., Vincent J., Duchateau N., Besson A., Dardevet M., Prodhomme D., Gibon Y., Hilbert G., Pailloux M. (2015). Transcriptional and Metabolic Alternations Rebalance Wheat Grain Storage Protein Accumulation under Variable Nitrogen and Sulfur Supply. Plant J..

[22] Fismes J., Vong P.C., Guckert A., Frossard E. (2000). Influence of Sulfur on Apparent N-Use Efficiency, Yield and Quality of Oilseed Rape (*Brassica napus* L.) Grown on a Calcareous Soil. Eur. J. Agron..

[23] Salvagiotti F., Castellarín J.M., Miralles D.J., Pedrol H.M. (2009). Sulfur Fertilization Improves Nitrogen Use Efficiency in Wheat by Increasing Nitrogen Uptake. Field Crops Res..

[24] Carciochi W.D., Divito G.A., Fernández L.A., Echeverría H.E. (2017). Sulfur Affects Root Growth and Improves Nitrogen Recovery and Internal Efficiency in Wheat. J. Plant Nutr..

[25] Tea I., Genter T., Violleau F., Kleiber D. (2005). Changes in the Glutathione Thiol-Disulfide Status in Wheat Grain by Foliar Sulphur Fertilization: Consequences for the Rheological Properties of Dough. J. Cereal Sci..

[26] Tea I., Genter T., Naulet N., Lummerzheim M., Kleiber D. (2007). Interaction between Nitrogen and Sulfur by Foliar Application and Its Effects on Flour Bread-Making Quality. J. Sci. Food Agric..

[27] Wilson T.L., Guttieri M.J., Nelson N.O., Fritz A., Tilley M. (2020). Nitrogen and Sulfur Effects on Hard Winter Wheat Quality and Asparagine Concentration. J. Cereal Sci..

[28] Guerrini L., Napoli M., Mancini M., Masella P., Cappelli A., Parenti A., Orlandini S. (2020). Wheat Grain Composition, Dough Rheology and Bread Quality as Affected by Nitrogen and Sulfur Fertilization and Seeding Density. Agronomy.

[29] Cai J., Zang F., Xin L., Zhou Q., Wang X., Zhong Y., Huang M., Dai T., Jiang D. (2022). Effects of Cysteine and Inorganic Sulfur Applications at Different Growth Stages on Grain Protein and End-Use Quality in Wheat. Foods.

[30] Wrigley C.W., Du Cros D.L., Fullington J.G., Kasarda D.D. (1984). Changes in Polypeptide Composition and Grain Quality Due to Sulfur Deficiency in Wheat. J. Cereal Sci..

[31] Swamy U., Wang M., Tripathy J.N., Kim S.-K., Hirasawa M., Knaff D.B., Allen J.P. (2005). Structure of Spinach Nitrite Reductase:  Implications for Multi-Electron Reactions by the Iron−Sulfur:Siroheme Cofactor. Biochemistry.

[32] Geng J., Ma Q., Chen J., Zhang M., Li C., Yang Y., Yang X., Zhang W., Liu Z. (2016). Effects of Polymer Coated Urea and Sulfur Fertilization on Yield, Nitrogen Use Efficiency and Leaf Senescence of Cotton. Field Crops Res..

[33] Tao Z., Chang X., Wang D., Wang Y., Ma S., Yang Y., Zhao G. (2018). Effects of Sulfur Fertilization and Short-Term High Temperature on Wheat Grain Production and Wheat Flour Proteins. Crop J..

[34] Steinfurth D., Zörb C., Braukmann F., Mühling K.H. (2012). Time-Dependent Distribution of Sulphur, Sulphate and Glutathione in Wheat Tissues and Grain as Affected by Three Sulphur Fertilization Levels and Late S Fertilization. J. Plant Physiol..

[35] Daher Al-Salami A.S., Hanoon Mohson K., Abdullhay Desher M. (2021). Effect of Agriculture Sulfur Fertilizer Levels on Growth, and Yield of Wheat (*Triticum aestivum* L.). Plant Arch..

[36] Doerge R.W. (2002). Mapping and Analysis of Quantitative Trait Loci in Experimental Populations. Nat. Rev. Genet..

[37] Cao W., Jia J., Jin J., Horst W.J., Schenk M.K., Bürkert A., Claassen N., Flessa H., Frommer W.B., Goldbach H., Olfs H.-W., Römheld V., Sattelmacher B. (2001). Identification and Interaction Analysis of QTL for Phosphorus Use Efficiency in Wheat Seedlings. Plant Nutrition: Food Security and Sustainability of Agro-Ecosystems Through Basic and Applied Research.

[38] Su J.-Y., Zheng Q., Li H.-W., Li B., Jing R.-L., Tong Y.-P., Li Z.-S. (2009). Detection of QTLs for Phosphorus Use Efficiency in Relation to Agronomic Performance of Wheat Grown under Phosphorus Sufficient and Limited Conditions. Plant Sci..

[39] Guo Y., Kong F., Xu Y., Zhao Y., Liang X., Wang Y., An D., Li S. (2012). QTL Mapping for Seedling Traits in Wheat Grown under Varying Concentrations of N, P and K Nutrients. Theor. Appl. Genet..

[40] Kong F.-M., Guo Y., Liang X., Wu C.-H., Wang Y.-Y., Zhao Y., Li S.-S. (2013). Potassium (K) Effects and QTL Mapping for K Efficiency Traits at Seedling and Adult Stages in Wheat. Plant Soil.

[41] Sun J., Guo Y., Zhang G., Gao M., Zhang G., Kong F., Zhao Y., Li S. (2013). QTL Mapping for Seedling Traits under Different Nitrogen Forms in Wheat. Euphytica.

[42] Zhao Y., Li X., Zhang S., Wang J., Yang X., Tian J., Hai Y., Yang X. (2014). Mapping QTLs for Potassium-Deficiency Tolerance at the Seedling Stage in Wheat (*Triticum aestivum* L.). Euphytica.

[43] Gong X.-P., Liang X., Guo Y., Wu C.-H., Zhao Y., Li X.-H., Li S.-S., Kong F.-M. (2015). Quantitative Trait Locus Mapping for Potassium Use Efficiency Traits at the Seedling Stage in Wheat under Different Nitrogen and Phosphorus Treatments. Crop Sci..

[44] Yuan Y., Zhang M., Zheng H., Kong F., Guo Y., Zhao Y., An Y. (2020). Detection of QTL for Phosphorus Efficiency and Biomass Traits at the Seedling Stage in Wheat. Cereal Res. Commun..

[45] Safdar L.B., Andleeb T., Latif S., Umer M.J., Tang M., Li X., Liu S., Quraishi U.M. (2020). Genome-Wide Association Study and QTL Meta-Analysis Identified Novel Genomic Loci Controlling Potassium Use Efficiency and Agronomic Traits in Bread Wheat. Front. Plant Sci..

[46] Lavoignat M., Cassan C., Pétriacq P., Gibon Y., Heumez E., Duque C., Momont P., Rincent R., Blancon J., Ravel C. (2024). Different Wheat Loci Are Associated to Heritable Free Asparagine Content in Grain Grown under Different Water and Nitrogen Availability. Theor. Appl. Genet..

[47] Peleg Z., Cakmak I., Ozturk L., Yazici A., Jun Y., Budak H., Korol A.B., Fahima T., Saranga Y. (2009). Quantitative Trait Loci Conferring Grain Mineral Nutrient Concentrations in Durum Wheat × Wild Emmer Wheat RIL Population. Theor. Appl. Genet..

[48] Fradgley N.S., Gardner K., Kerton M., Swarbreck S.M., Bentley A.R. (2022). Trade-Offs in the Genetic Control of Functional and Nutritional Quality Traits in UK Winter Wheat. Heredity.

[49] Li Z., Ni Z., Peng H., Liu Z., Nie X., Xu S. (2007). Molecular Mapping of QTLs for Root Response to Phosphorus Deficiency at Seedling Stage in Wheat (*Triticum aestivum* L.). Prog. Nat. Sci..

[50] Wang Y., Sun X., Zhao Y., Kong F., Guo Y., Zhang G., Pu Y., Wu K., Li S. (2011). Enrichment of a Common Wheat Genetic Map and QTL Mapping for Fatty Acid Content in Grain. Plant Sci..

[51] Hoagland D.R., Arnon D.I. (1938). The Water Culture Method for Growing Plant Without Soil.

[52] Knapp S.J., Stroup W.W., Ross W.M. (1985). Exact Confidence Intervals for Heritability on a Progeny Mean Basis. Crop Sci..

[53] Wang S., Basten C., Gaffney P., Zeng Z. (2007). Windows QTL Cartographer 2.0.

[54] Churchill G.A., Doerge R.W. (1994). Empirical Threshold Values for Quantitative Trait Mapping. Genetics.

[55] Stoll M., Kwitek-Black A.E., Cowley A.W., Harris E.L., Harrap S.B., Krieger J.E., Printz M.P., Provoost A.P., Sassard J., Jacob H.J. (2000). New Target Regions for Human Hypertension via Comparative Genomics. Genome Res..

[56] Tsujimoto Y., Yamamoto Y., Hayashi K., Zakaria A.I., Inusah Y., Hatta T., Fosu M., Sakagami J.-I. (2013). Topographic Distribution of the Soil Total Carbon Content and Sulfur Deficiency for Rice Cultivation in a Floodplain Ecosystem of the Northern Region of Ghana. Field Crops Res..

[57] Sharma V., Rena V., Kumar D., Pandey R.N., Singh B. (2016). Sulfur Regulates Iron Uptake and Iron Use Efficiency in Bread and Durum Wheat. Indian J. Plant Physiol..

[58] Zhang H., Wang H. (2015). QTL Mapping for Traits Related to P-Deficient Tolerance Using Three Related RIL Populations in Wheat. Euphytica.

[59] Zhang M., Gao M., Zheng H., Yuan Y., Zhou X., Guo Y., Zhang G., Zhao Y., Kong F., An Y. (2019). QTL Mapping for Nitrogen Use Efficiency and Agronomic Traits at the Seedling and Maturity Stages in Wheat. Mol. Breed..

[60] An D., Su J., Liu Q., Zhu Y., Tong Y., Li J., Jing R., Li B., Li Z. (2006). Mapping QTLs for Nitrogen Uptake in Relation to the Early Growth of Wheat (*Triticum aestivum* L.). Plant Soil.

[61] Laperche A., Devienne-Barret F., Maury O., Le Gouis J., Ney B. (2006). A Simplified Conceptual Model of Carbon/Nitrogen Functioning for QTL Analysis of Winter Wheat Adaptation to Nitrogen Deficiency. Theor. Appl. Genet..

[62] Cui F., Fan X., Zhao C., Zhang W., Chen M., Ji J., Li J. (2014). A Novel Genetic Map of Wheat: Utility for Mapping QTL for Yield under Different Nitrogen Treatments. BMC Genet..

[63] Collins N.C., Tardieu F., Tuberosa R. (2008). Quantitative Trait Loci and Crop Performance under Abiotic Stress: Where Do We Stand?. Plant Physiol..

[64] Ninkuu V., Zhou Y., Liu H., Sun S., Liu Z., Liu Y., Yang J., Hu M., Guan L., Sun X. (2024). Regulation of Nitrogen Metabolism by *COE2* under Low Sulfur Stress in *Arabidopsis*. Plant Sci..

[65] Forieri I., Aref R., Wirtz M., Hell R. (2021). Micrografting Provides Evidence for Systemic Regulation of Sulfur Metabolism between Shoot and Root. Plants.

