# Supplementary material for: Quantitative Trait Loci Mappings for the Sulfur Utilization Efficiency-Related Traits at the Seedling Stage of Wheat
Source: Genes (Basel). 2024 Nov 29;15(12):1550. doi: 10.3390/genes15121550 (PMC11727847; doi:10.3390/genes15121550)
Supplement: Supplementary file 1 [file genes-15-01550-s001.zip › genes-3309331-supplementary.pdf]

Supplementary Materials

Table S1 Nutrient solution ingredients for wheat seedling growth.

Table S2 Phenotypic analysis of seedling traits for RIL population under different S treatments.

Table S3 ANOVA for 13 S utilization efficiency related traits.

Table S4 Correlation coefficient of seedling traits for RIL population.

Table S5 Additive QTLs for the investigated traits in the three environments and their mean values (MV).

Table S6 Locations of QTL clusters of S-related traits in this paper and the other traits in previous studies.

Table S1 Nutrient solution ingredients for wheat seedling growth.

Treatments	Ingredients	Concentration (mmol/L)	Ingredient	Concentration (μmol/L)
T1 (0.1S)	KCl	1.5	H_3BO_3	1.0
	$CaCl_2$	1.5	$MnSO_4 \cdot H_2O$	1.0
	KH_2PO_4	0.2	$CuSO_4 \cdot 5H_2O$	0.5
	$MgSO_4 \cdot 7H_2O$	0.1	$ZnSO_4 \cdot 7H_2O$	1.0
	MgCl	0.4	Fe-EDTA	100
	NH_4Cl	2.0	$(NH_4)_6Mo_7O_{24} \cdot 4H_2O$	0.1
	$Ca(NO_3)_2 \cdot 4H_2O$	1.0		
T2 (0.5S)	KCl	1.5	H_3BO_3	1.0
	$CaCl_2$	1.5	$MnSO_4 \cdot H_2O$	1.0
	KH_2PO_4	0.2	$CuSO_4 \cdot 5H_2O$	0.5
	$MgSO_4 \cdot 7H_2O$	0.5	$ZnSO_4 \cdot 7H_2O$	1.0
	NH_4Cl	2.0	Fe-EDTA	100
	$Ca(NO_3)_2 \cdot 4H_2O$	1.0	$(NH_4)_6Mo_7O_{24} \cdot 4H_2O$	0.1
T3 (1.5S)	KCl	1.5	H_3BO_3	1.0
	$CaCl_2$	1.5	$MnSO_4 \cdot H_2O$	1.0
	KH_2PO_4	0.2	$CuSO_4 \cdot 5H_2O$	0.5
	$MgSO_4 \cdot 7H_2O$	0.5	$ZnSO_4 \cdot 7H_2O$	1.0
	$(NH_4)_2SO_4 \cdot H_2O$	1.0	Fe-EDTA	100
	$Ca(NO_3)_2 \cdot 4H_2O$	1.0	$(NH_4)_6Mo_7O_{24} \cdot 4H_2O$	0.1

Table S2 Phenotypic analysis of seedling traits for RIL population under different S treatments.

Traits[a]	Treatments	Shannong 483[b]	Chuan 35050	MIN	MAX	AV[c]	SD	CV(%)	h_B^2(%)
SH	L	35.63**	32.34	27.97	43.03	34.78b	2.87	8.26	77.33
(cm)	M	37.93	35.07	31.42	48.64	38.66a	3.08	7.96	
	H	36.69	35.49	31.13	49.00	38.58a	3.07	7.95	
MRL	L	22.65**	18.18	13.59	25.44	18.77b	2.70	14.36	65.97
(cm)	M	22.00**	19.20	14.63	26.41	18.79b	2.63	13.98	
	H	22.94**	17.59	14.45	27.22	20.70a	2.88	14.76	
RN	L	8.63	7.81	5.58	11.50	8.27a	1.13	13.69	62.32
	M	8.34	6.51	6.11	11.13	7.74b	0.96	12.39	
	H	9.63	9.38	5.94	11.50	8.54a	1.20	14.10	
RDW	L	37.41	35.12	20.32	57.66	35.03a	7.67	21.88	66.57
(mg·plant^{-1})	M	37.83	28.76	22.53	61.07	34.10a	7.52	22.05	
	H	33.86	37.30	19.82	55.57	33.54a	7.31	21.80	
SDW	L	162.50	147.29	92.17	223.56	140.02a	27.22	19.44	68.62
(mg·plant^{-1})	M	184.94	120.36	89.35	230.50	143.22a	28.10	19.62	
	H	160.25	156.01	84.94	229.50	146.45a	28.19	19.25	
TDW	L	199.91	182.41	112.49	281.22	175.14a	34.76	19.85	68.19
(mg·plant^{-1})	M	222.77	149.11	112.81	291.57	177.52a	34.83	19.62	
	H	194.11	193.31	104.75	285.07	179.99a	34.48	19.16	
RSR	L	0.23	0.24	0.17	0.34	0.25a	0.03	12.19	64.19
	M	0.20	0.24	0.18	0.34	0.24b	0.03	12.12	
	H	0.21	0.24	0.17	0.32	0.23c	0.03	12.35	
RSC	L	0.07	0.07	0.04	0.13	0.07b	0.02	28.37	68.52
(mg·plant^{-1})	M	0.09	0.06	0.05	0.15	0.08a	0.02	26.85	
	H	0.08	0.09	0.04	0.15	0.08a	0.02	27.78	
SSC	L	0.41	0.32	0.21	0.61	0.33b	0.08	25.46	71.05
(mg·plant^{-1})	M	0.49	0.29	0.18	0.60	0.34b	0.09	25.80	
	H	0.43	0.37	0.19	0.62	0.37a	0.09	24.22	
TSC	L	0.48	0.39	0.24	0.71	0.40c	0.10	24.93	72.35
(mg·plant^{-1})	M	0.58	0.35	0.23	0.73	0.42b	0.11	25.09	
	H	0.51	0.46	0.24	0.72	0.45a	0.11	23.96	
RSUE	L	21.95	19.36	9.67	27.61	18.53a	4.79	25.83	54.99
(mg^{2}RDW·μg^{-1}RSC)	M	16.75	13.60	9.23	25.38	15.11b	3.65	24.16	
	H	13.86	16.11	7.45	25.29	14.19b	3.17	22.34	
SSUE	L	64.44	68.03	39.97	108.79	61.65a	12.59	20.42	56.61
(mg^{2}RDW·μg^{-1}SSC)	M	70.93*	50.22	38.84	96.10	61.20a	12.08	19.75	
	H	60.00	65.77	32.77	95.34	59.35a	11.87	20.01	
TSUE	L	83.54	86.40	49.23	146.94	79.44a	16.80	21.14	55.22
(mg^{2}RDW·μg^{-1}TSC)	M	87.20	63.64	48.59	121.49	75.99ab	14.57	19.17	
	H	73.45	81.84	42.84	114.26	73.14b	13.81	18.88	

NOTE: [a] SH, Shoot height per plant; MRL, Maximum root length per plant; RN, Root number per plant; RDW, Root dry weight per plant; SDW, Shoot dry weight per plant; TDW, Total dry weight per plant; RSR, Root-shoot ratio; RSC, Root sulfur content per plant; SSC, Shoot sulfur content per plant; TSC, total sulfur content per plant; RSUE, Root sulfur utilization efficiency; SSUE, shoot sulfur utilization efficiency; TSUE, total sulfur utilization efficiency; LS: low S; MS: moderate S; HS: high S.

[b] * and ** mean significant differences at $p < 0.05$ and 0.01, respectively.

[c] Means in the same column followed by the same letter do not differ significantly according to the LSD test (letter indicating $p = 0.05$).

Table S3 ANOVA for 13 S utilization efficiency related traits.

Trait	Genotype	Treatment	replication
SH	39.90**	41.30**	1526.90**
MRL	47.80**	1229.70**	1859.20**
RN	5.60**	40.60**	1896.90**
RDW	290.00**	156.90	839.00**
SDW	405.00**	2515.00*	29992.00**
TDW	6282.00**	1373.00	20035.00**
RSR	0.004**	0.030**	0.25409**
RSC	0.002**	0.012**	0.0005
SSC	0.038**	0.116**	0.723**
TSC	0.056**	0.196**	0.686**
RSUE	67.50**	1355.20**	522.20**
SSUE	703.10**	420.80	92.00
TSUE	1062.70**	2656.90**	1267.10*

*, and ** indicate the significance at $p \leqslant 0.05$ and $p \leqslant 0.01$, respectively.

Table S4 Correlation coefficient of seedling traits for RIL population.

	SH	MRL	RN	RDW	SDW	TDW	RSR	RSC	SSC	TSC	RSUE	SSUE
MRL	0.408**											
RN	0.466**	0.006										
RDW	0.444**	0.340**	0.748**									
SDW	0.626**	0.252**	0.737**	0.845**								
TDW	0.599**	0.279**	0.759**	0.904**	0.991**							
RSR	-0.724**	0.208*	0.122	0.406**	0.125	0.008						
RSC	0.501**	0.369**	0.615**	0.898**	0.829**	0.869**	0.254**					
SSC	0.545**	0.352**	0.687**	0.805**	0.911**	0.910**	0.043	0.841**				
TSC	0.550**	0.365**	0.691**	0.844**	0.919**	0.926**	0.015	0.894**	0.994**			
RSUE	0.245**	0.231**	0.704**	0.860**	0.633**	0.701**	0.502**	0.553**	0.551**	0.566**		
SSUE	0.577**	0.058	0.617**	0.676**	0.860**	0.842**	-0.209*	0.601**	0.577**	0.597**	0.569**	
TSUE	0.532**	0.110	0.683**	0.782**	0.870**	0.879**	0.040	0.650**	0.613**	0.636**	0.718**	0.971**

Note：SH, Shoot height per plant; MRL, Maximum root length per plant; RN, Root number per plant; RDW, Root dry weight per plant; SDW, Shoot dry weight per plant; TDW, Total dry weight per plant; RSR, Root-shoot ratio; RSC, Root S content per plant; SSC, Shoot S content per plant; TSC, total S content per plant; RSUE, Root S utilization efficiency; SSUE, shoot S utilization efficiency; TSUE, total S utilization efficiency.

*and**indicate significant correlation at 0.05 and 0.01 levels, respectively

Table S5 Additive QTLs for the investigated traits in the three environments and their mean values (MV).

Trait[a]	QTL	Enviroment	Marker interval	Site[b] (cM)	LOD	Additive Effect	R^2(%)
SH	*QSh.1-1A*	T3AV	*wPt731490-wPt8455*	2	4.2	1.08	11.7
	QSh.2-1A	T1AV	*wPt731476-gwm99*	12	3.5	1.22	8.0
		T2E1	*wPt731476-gwm99*	10	5.3	2.81	21.1
	QSh-1D	T1E1	*wmc432b-wPt665480*	6	8.5	-2.14	26.5
		T1AV	*wPt4647-swes1100*	2	3.3	-0.97	10.9
		T2E1	*wmc432b-wPt665480*	5	5.2	-1.60	15.1
		T2AV	*wmc432b-wPt665480*	4	5.2	-1.25	14.7
		T3E1	*wmc432b-wPt665480*	1	7.1	-1.81	20.1
	QSh.1-2B	T1AV	*wmc154b-wPt7970*	4	4.6	1.39	19.8
	QSh.2-2B	T1E1	*wPt9736-wPt5502*	2	3.8	2.02	12.2
	QSh-3A	T2E1	*wPt4692-ubc859e*	34	4.0	2.02	18.7
		T2E2	*wPt4692-ubc859e*	0	4.3	1.26	9.8
		T2AV	*wPt4692-ubc859e*	3	7.3	1.46	21.3
	QSh.1-3B	T1AV	*barc139-srap2*	4	3.3	-0.84	7.8
		T2E2	*barc139-srap2*	0	7.7	-1.79	18.2
		T3AV	*swes862-barc139*	5	3.8	-0.98	9.6
	QSh.2-3B	T2AV	*wmc3a-wPt7229*	13	4.9	-1.23	14.4
	QSh-4B	T3E2	*swes24c-wPt7569*	17	3.1	-1.46	10.4
		T3AV	*swes24c-wPt7569*	16	3.1	-1.06	10.3
	QSh-6A	T3E2	*wPt668031-wPt4229*	5	3.1	1.22	8.3
		T3AV	*wPt3247-wPt1695*	1	3.5	1.00	9.5
	QSh.1-6B	T2E2	*wPt5176-wPt1541*	0	6.1	1.52	14.1
		T2AV	*ubc840b-wPt5176*	10	5.7	1.18	13.8
	QSh.2-6B	T1E2	*swes199-swes181*	1	4.1	-1.24	11.5
		T1AV	*swes199-swes181*	2	3.6	-1.00	10.2
	QSh-7A	T3AV	*swes134b-ubc822b*	10	3.5	1.17	14.5
MRL	*QMrl.1-1A*	T3E2	*wPt669607-wPt665590*	27	4.8	-1.20	9.7
	QMrl.2-1A	T3AV	*wmc93a-wmc312b*	1	5.5	-1.12	12.3
	QMrl.1-2D	T1E2	*wPt2544-wPt730613*	5	8.8	-1.39	22.0
		T3AV	*wPt9749-wPt671778*	2	5.2	-1.05	11.3
		T2E2	*wPt667476-wPt5865*	1	10.3	-1.77	23.1
		T2AV	*wPt667476-wPt5865*	0	6.8	-1.02	14.0
		T3E2	*wPt2781-trap4d*	4	8.0	-1.82	21.4
	QMrl.2-2D	T2E1	*ubc815b-wmc445b*	1	4.8	-1.26	13.9
	QMrl-3A	T1E1	*wPt664829-wPt731120*	13	4.5	-1.32	12.4
		T2E1	*wPt666438-wPt4692*	7	4.2	-1.28	16.1
	QMrl.1-3B	T1AV	*wPt5836-wPt664393*	3	8.8	-1.43	25.4
		T3E1	*wPt5836-wPt664393*	1	7.9	-1.48	21.0
	QMrl.2-3B	T2AV	*wPt7229-barc164*	3	3.7	-0.78	8.0
	QMrl.1-4D	T3AV	*gwm624-gwm609*	0	4.3	0.97	9.8
	QMrl.2-4D	T1E1	*wPt4572-wPt2379*	12	6.6	-2.39	22.5
	QMrl-5D	T2AV	*swes342a-swes342b*	0	3.0	-0.65	5.1
	QMrl-6A	T1E2	*wPt4229-wPt731002*	0	8.3	1.41	18.5
	QMrl-6D	T1AV	*wPt667726-wPt668152*	8	3.7	-0.83	9.3
		T2AV	*barc21a-wPt667006*	5	3.8	-0.78	8.0
		T3AV	*wPt667006-wPt667726*	0	6.5	-1.10	12.8
	QMrl.1-7B	T1AV	*swes19-wPt6156*	16	3.7	1.15	13.6
		T2AV	*swes19-wPt6156*	17	4.9	1.28	17.0
	QMrl.2-7B	T2E2	*wPt1066-wPt0194*	6	3.6	1.58	9.3
RN	*QRn-1A*	T2E2	*wPt5316-wPt666963*	0	3.1	0.45	8.5
	QRn-1D	T1E1	*wPt665480-wPt666067*	4	5.8	-0.72	18.3
		T1AV	*wPt665480-wPt666067*	6	3.9	-0.43	13.9

Note: [a] SH, Shoot height per plant; MRL, Maximum root length per plant; RN, Root number per plant; RDW, Root dry weight per plant; SDW, Shoot dry weight per plant; TDW, Total dry weight per plant; RSR, Root-shoot ratio; RSC, Root sulfur content per plant; SSC, Shoot sulfur content per plant; TSC, total sulfur content per plant; RSUE, Root sulfur utilization efficiency; SSUE, shoot sulfur utilization efficiency; TSUE, total sulfur utilization efficiency.

[b] Site is the distance of LOD peak value for QTL after the first marker in the marker interval.

Table S5 Continued

Trait[a]	QTL	Enviroment	Marker interval	Site[b] (cM)	LOD	Additive Effect	R^2(%)
		T2E1	*wPt665480-wPt666067*	2	4.2	-0.53	13.1
		T2AV	*wmc336b-wmc432b*	1	4.6	-0.40	15.6
		T3E1	*wPt665480-wPt666067*	2	4.5	-0.48	13.0
	QRn.1-2B	T3E2	*wmc154a-wmc154b*	1	4.2	0.64	13.0
		T3AV	*wPt5374-wmc154a*	12	7.3	0.64	22.5
	QRn.2-2B	T1E2	*srap1a-gwm120*	1	3.4	-0.46	10.1
	QRn-3B	T2E1	*wPt2416-issr25a*	4	3.5	-0.52	11.8
	QRn-4A	T3E2	*wPt4620-wPt5172*	0	3.2	-0.52	8.3
		T3AV	*wPt4620-wPt5172*	0	5.7	-0.53	14.9
	QRn-4B	T1E2	*wPt3991-wPt5334*	0	5.9	-0.52	17.2
	QRn-5B	T1E1	*gwm261b-gwm234*	4	3.7	0.96	12.3
	QRn.1-5D	T2AV	*swes558b-swes558a*	1	3.8	0.32	10.5
	QRn.2-5D	T2E2	*barc49b-barc49a*	2	5.2	0.51	16.8
	QRn-6A	T3AV	*wPt671561-wPt664603*	0	3.5	0.37	8.4
	QRn.1-7A	T1E2	*wPt668064-wPt1510*	0	3.9	-0.40	11.1
		T2E2	*wPt667328-wPt668064*	0	3.1	-0.29	8.4
	QRn.2-7A	T1E1	*wPt4637-barc121*	0	4.8	-0.62	11.8
	QRn.3-7A	T1E1	*swes430b-srap15*	7	3.6	-0.59	9.0
		T3E1	*swes430b-srap15*	7	3.2	-0.46	9.0
RDW	*QRdw-1D*	T1E1	*wmc432b-wPt665480*	6	5.4	-3.91	18.2
		T2E1	*wmc432b-wPt665480*	9	4.5	-3.34	16.5
		T3E1	*wmc432b-wPt665480*	9	5.5	-3.56	16.9
		T3AV	*wPt665480-wPt666067*	0	3.6	-2.38	9.5
	QRdw-2A	T1E2	*swes217b-gwm71b*	4	5.9	-7.40	16.7
		T1AV	*swes217b-gwm71b*	4	3.4	-2.60	9.0
	QRdw-3A	T2E1	*wPt4725-wPt1562*	5	3.3	-4.15	10.3
	QRdw-3B	T2E2	*wPt7614-wPt5072*	0	3.3	3.60	8.0
	QRdw-6A	T1E1	*wPt731524-wPt7204*	8	3.8	2.90	10.5
		T1E2	*wPt4229-wPt731002*	0	7.1	5.33	19.1
		T1AV	*wPt668031-wPt4229*	6	8.0	3.80	23.3
		T2E2	*wPt3247-wPt1695*	0	7.7	5.88	20.6
		T2AV	*wPt3247-wPt1695*	2	4.1	2.86	13.8
		T3E2	*wPt3247-wPt1695*	2	4.2	4.53	14.5
		T3AV	*wPt672030-wPt671568*	4	4.4	3.01	15.1
	QRdw-6B	T3E2	*swes1106a-wPt8015*	0	3.4	-3.56	9.2
	QRdw-7A	T3E1	*swes430b-srap15*	7	3.6	-3.00	10.0
SDW	*QSdw-1A*	T3AV	*wPt9708-wPt6654*	3	3.2	13.00	8.4
	QSdw.1-1B	T2E1	*wmc128-swes189*	3	3.4	-11.44	8.5
		T2AV	*wmc128-swes189*	2	3.7	-10.08	9.3
	QSdw.2-1B	T2E1	*wmc314-ubc834b*	8	3.4	10.99	8.3
		T2AV	*ubc834b-ubc880d*	0	3.8	10.18	9.3
	QSdw-1D	T1E1	*wmc432b-wPt665480*	11	6.4	-14.93	18.1
		T2E1	*wmc432b-wPt665480*	11	5.6	-13.60	15.5
		T2AV	*wPt666067-wPt4647*	7	4.0	-9.90	11.9
		T3E1	*wmc432b-wPt665480*	11	9.4	-20.52	21.6
		T3AV	*wmc432b-wPt665480*	10	3.7	-9.62	9.8
	QSdw-2A	T1E2	*gwm71b-gwm71c*	0	6.9	-25.06	17.6
		T1AV	*swes217b-gwm71b*	4	3.4	-9.04	8.4
		T2E2	*gwm71b-gwm71c*	3	3.3	-11.43	7.4
	QSdw-2B	T3E2	*wPt5374-wmc154a*	12	5.7	15.22	13.4
	QSdw-3B	T2E1	*wPt2416-issr25a*	6	4.4	-12.58	13.1
	QSdw.1-4A	T3E2	*wPt0032-wPt4620*	3	3.5	-11.42	7.7
		T3AV	*wPt0032-wPt4620*	2	3.2	-8.70	7.9
	QSdw.2-4A	T3E1	*wPt671707-barc70a*	2	3.9	-12.16	10.8
	QSdw-4B	T2E2	*wPt7569-wPt3991*	5	3.9	-14.67	12.3
	QSdw-6A	T1E2	*wPt4229-wPt731002*	0	8.2	18.62	21.7
		T1AV	*wPt4229-wPt731002*	1	5.5	11.04	15.3

Table S5 Continued

Trait[a]	QTL	Enviroment	Marker interval	Site[b] (cM)	LOD	Additive Effect	R^2(%)
		T2E2	*wPt7204-swes1062*	0	9.7	19.55	21.0
		T2AV	*wPt4229-wPt731002*	2	6.0	12.26	17.6
		T3E2	*wPt668031-wPt4229*	5	6.7	22.48	18.2
		T3AV	*wPt4229-wPt731002*	0	3.7	8.66	8.7
	QSdw-6B	T3E1	*wPt8412-wmc487*	11	4.5	13.02	11.2
	QSdw-7A	T1E1	*wPt4637-barc121*	0	3.9	-11.71	9.6
TDW	*QTdw.1-1B*	T2E1	*wmc128-swes189*	2	4.0	-16.11	10.8
		T2AV	*wmc128-swes189*	3	3.6	-12.22	9.1
	QTdw.2-1B	T2AV	*ubc834b-ubc880d*	0	4.1	13.06	10.4
	QTdw.3-1B	T2E1	*swes119a-ubc857a*	12	3.3	12.67	9.0
	QTdw-1D	T1E1	*wmc432b-wPt665480*	11	8.0	-20.98	23.8
		T2E1	*wmc432b-wPt665480*	9	6.3	-18.76	20.0
		T2AV	*wmc432b-wPt665480*	12	4.6	-12.86	12.6
		T3E1	*wmc432b-wPt665480*	11	9.3	-24.55	28.4
		T3AV	*wmc432b-wPt665480*	8	3.6	-11.95	10.8
	QTdw-2A	T1E2	*gwm71b-gwm71c*	0	6.8	-32.94	17.3
		T1AV	*swes217b-gwm71b*	5	3.8	-11.84	9.2
		T2E2	*gwm71b-gwm71c*	3	3.6	-15.55	8.4
	QTdw-2B	T3E2	*wPt5374-wmc154a*	10	4.3	17.40	11.4
	QTdw-3A	T1E1	*wPt668205-swes1157*	2	3.8	23.48	13.7
	QTdw-3B	T2E1	*wPt2416-issr25a*	6	3.6	-13.82	10.7
	QTdw.1-4A	T3E2	*wPt0032-wPt4620*	1	3.7	-15.14	8.6
		T3AV	*wPt0032-wPt4620*	2	3.0	-10.53	7.7
	QTdw.2-4A	T1E1	*wPt671707-barc70a*	0	3.2	-12.38	8.0
		T3E1	*wPt671707-barc70a*	3	3.5	-14.07	10.1
	QTdw-4B	T2E2	*wPt7569-wPt3991*	4	3.7	-17.88	12.1
	QTdw-6A	T1E2	*wPt4229-wPt731002*	0	8.5	24.66	22.3
		T1AV	*wPt7204-swes1062*	0	7.9	15.77	19.8
		T2E2	*wPt7204-swes1062*	0	8.1	22.36	20.7
		T2AV	*wPt3247-wPt1695*	2	6.9	16.16	19.9
		T3E2	*wPt668031-wPt4229*	7	7.2	27.54	17.9
		T3AV	*wPt4229-wPt731002*	1	5.3	13.85	15.0
	QTdw-6B	T3E1	*wPt8412-wmc487*	11	4.2	15.14	10.7
	QTdw-7A	T1E1	*wPt4637-barc121*	0	5.2	-17.05	13.1
RSR	*QRsr-1A*	T1E2	*wPt730172-wPt669607*	0	5.1	-0.03	14.6
	QRsr-1D	T2AV	*wPt7946-wmc336b*	19	3.5	-0.01	9.3
	QRsr.1-2B	T3E2	*wPt3561-wPt5374*	-1	6.2	-0.01	9.4
	QRsr.2-2B	T1E1	*wPt6223-wPt8460*	0	3.7	-0.01	9.4
		T3E1	*wPt6158-wPt6223*	0	3.9	-0.01	9.8
		T3AV	*wPt5587-wPt0643*	0	6.4	-0.01	15.9
	QRsr.3-2B	T2E2	*srap1a-gwm120*	6	4.4	-0.02	15.0
		T2AV	*srap1a-gwm120*	6	3.2	-0.01	11.2
	QRsr.1-3A	T1E2	*wPt4725-wPt1562*	0	3.1	0.02	8.3
	QRsr.2-3A	T1E1	*ubc859e-wPt730892*	8	3.1	0.02	14.9
	QRsr.1-3B	T1AV	*swes862-barc139*	1	4.4	0.01	12.7
	QRsr.2-3B	T2AV	*wPt0751-wPt7614*	3	3.1	0.01	9.4
		T3AV	*wPt4412-wPt2416*	0	4.2	0.01	9.8
	QRsr.3-3B	T1E2	*gwm547-gwm340*	12	4.6	0.02	13.0
	QRsr-4A	T2AV	*wPt671707-barc70a*	4	3.0	0.01	9.2
	QRsr-5A	T2E2	*trap3-issr22c*	1	4.0	-0.02	12.0
		T3E1	*trap3-issr22c*	22	3.1	-0.01	7.7
	QRsr-5B	T3AV	*wPt1302-wPt6498*	4	3.9	0.01	9.5
	QRsr.1-6A	T1E1	*wPt671558-ubc810a*	0	3.1	-0.01	8.0
	QRsr.2-6A	T3E1	*swes119b-wmc163*	2	6.5	0.02	20.5
	QRsr.3-6A	T3AV	*wPt664603-wPt730711*	10	3.2	0.01	10.3
	QRsr.4-6A	T3E2	*ubc860a-swes123b*	0	3.3	0.01	8.5
	QRsr-7A	T2E1	*ubc811a-swes134a*	2	3.8	0.01	12.2

Table S5 Continued

Trait[a]	QTL	Enviroment	Marker interval	Site[b] (cM)	LOD	Additive Effect	R^2(%)
RSC	*QRsc-1A*	T3E1	*wPt731490-wPt8455*	7	3.5	0.01	9.7
	QRsc-1B	T2E1	*ubc853b-ubc842*	22	3.7	0.01	10.5
		T2AV	*ubc842-wmc314*	0	3.4	0.01	8.7
	QRsc-1D	T1E1	*wmc432b-wPt665480*	6	5.8	-0.01	18.0
		T1AV	*wmc432b-wPt665480*	9	3.0	-0.01	8.9
		T3AV	*wPt7946-wmc336b*	20	3.9	-0.01	10.0
	QRsc-2A	T2E2	*gwm71b-gwm71c*	0	3.2	-0.01	8.0
		T3E2	*gwm71b-gwm71c*	0	3.6	-0.01	8.7
	QRsc-2B	T1E1	*wPt7970-wPt6805*	8	5.3	0.01	14.3
		T1AV	*wPt7970-wPt6805*	7	3.7	0.01	9.7
	QRsc-2D	T3E2	*wPt3757-wPt667054*	0	7.1	-0.02	18.7
	QRsc.1-3B	T3E1	*srap2-trap9*	4	4.1	-0.01	11.9
	QRsc.2-3B	T3AV	*wPt1940-wmc418*	4	3.6	-0.01	12.1
	QRsc.3-3B	T2E1	*wPt2416-issr25a*	6	3.4	-0.01	11.5
	QRsc-5B	T2E1	*gwm234-wPt1302*	2	3.0	0.02	27.2
		T2AV	*gwm234-wPt1302*	0	3.3	0.01	9.3
	QRsc-6A	T1E2	*wPt3247-wPt1695*	0	4.1	0.01	10.4
		T1AV	*wPt3247-wPt1695*	1	5.4	0.01	14.3
		T2E2	*wPt666494-wPt3247*	4	9.3	0.02	27.5
		T3E2	*wPt4229-wPt731002*	0	7.4	0.01	19.3
		T3AV	*wPt672030-wPt671568*	4	6.4	0.01	19.2
	QRsc-6B	T3E2	*wPt4924-wPt3060*	5	3.5	-0.01	11.9
	QRsc-7A	T3AV	*wPt4172-wPt8149*	0	4.9	0.01	12.0
	QRsc-7B	T2E1	*wPt4025-wPt664593*	3	4.4	0.02	24.9
SSC	*QSsc.1-1A*	T1AV	*wPt8455-wPt669484*	0	4.4	0.03	10.2
	QSsc.2-1A	T3AV	*wPt731476-gwm99*	0	3.3	-0.04	6.6
	QSsc-1D	T1E1	*wPt665480-wPt666067*	2	4.6	-0.04	12.7
		T2E1	*wmc432b-wPt665480*	11	5.3	-0.05	16.6
		T2AV	*wPt665480-wPt666067*	0	3.5	-0.03	8.4
		T3E1	*wPt665480-wPt666067*	0	4.3	-0.04	11.0
	QSsc-2A	T1E2	*gwm71b-gwm71c*	2	4.5	-0.04	11.0
		T2E2	*srap5b-barc220*	0	3.0	-0.03	6.4
	QSsc-2B	T3E2	*wPt5374-wmc154a*	7	6.7	0.06	20.7
		T3AV	*wmc154a-wmc154b*	2	4.0	0.03	20.1
	QSsc-3B	T1E1	*srap2-trap9*	4	3.5	-0.03	9.5
		T1AV	*trap9-ubc853c*	0	3.6	-0.03	8.1
		T2E1	*trap9-ubc853c*	0	3.1	-0.03	7.7
	QSsc-4A	T3E2	*wPt4620-wPt5172*	0	4.1	-0.04	9.3
	QSsc-6A	T1E1	*wPt672030-wPt671568*	2	3.7	0.03	10.1
		T1E2	*wPt668031-wPt4229*	6	5.9	0.04	15.0
		T1AV	*wPt672030-wPt671568*	3	7.2	0.04	20.0
		T2E2	*wPt668031-wPt4229*	1	7.2	0.05	17.1
		T2AV	*wPt3247-wPt1695*	0	4.5	0.03	10.8
		T3E2	*wPt666964-wPt666494*	0	3.5	0.04	7.8
		T3AV	*wPt3247-wPt1695*	0	4.2	0.03	9.1
	QSsc-6B	T1E2	*wPt8412-wmc487*	10	3.1	-0.03	7.2
	QSsc.1-7A	T3AV	*swes134b-ubc822b*	22	3.6	0.03	13.3
	QSsc.2-7A	T2E1	*barc121-ubc873c*	4	3.7	-0.04	11.1
	QSsc-7B	T2E2	*swes927b-swes624d*	0	5.9	-0.04	13.2
TSC	*QTsc.1-1A*	T1E1	*wPt8455-wPt669484*	0	3.2	0.03	7.2
		T3E1	*wPt8455-wPt669484*	0	3.1	0.04	8.1
	QTsc.2-1A	T3AV	*wPt731476-gwm99*	0	3.6	-0.05	8.3
	QTsc.1-1B	T2E1	*swes189-wmc419b*	0	3.6	-0.05	8.8
	QTsc.2-1B	T2E2	*ubc853b-ubc842*	18	3.2	0.05	10.7
	QTsc-1D	T1E1	*wmc432b-wPt665480*	11	5.9	-0.05	15.5
		T2E1	*wmc432b-wPt665480*	11	5.6	-0.05	15.4
		T2AV	*wmc432b-wPt665480*	11	3.8	-0.04	10.7

Table S5 Continued

Trait[a]	QTL	Enviroment	Marker interval	Site[b] (cM)	LOD	Additive Effect	R^2(%)
		T3E1	*wmc432b-wPt665480*	11	3.6	-0.04	10.3
		T3AV	*wmc432b-wPt665480*	11	4.1	-0.04	10.5
	QTsc-2A	T1E2	*gwm71b-gwm71c*	3	5.0	-0.05	11.7
		T2E2	*gwm71b-gwm71c*	0	4.6	-0.05	9.8
		T2AV	*swes598-barc15*	0	3.1	-0.03	7.0
	QTsc-2B	T3E2	*wPt5374-wmc154a*	8	4.7	0.07	16.3
		T3AV	*wPt5374-wmc154a*	10	3.1	0.04	9.8
	QTsc-3B	T1E1	*srap2-trap9*	3	6.2	-0.05	16.0
	QTsc-4B	T2E2	*wPt7569-wPt3991*	2	3.8	-0.05	11.3
	QTsc-5A	T1E2	*swes332b-swes1035b*	6	3.7	0.05	11.5
	QTsc-6A	T1E1	*wPt731524-wPt7204*	8	4.0	0.04	9.6
		T1E2	*wPt3247-wPt1695*	2	6.9	0.05	18.2
		T1AV	*wPt672030-wPt671568*	3	6.0	0.04	17.4
		T2E2	*wPt668031-wPt4229*	3	7.7	0.06	19.4
		T2AV	*wPt3247-wPt1695*	2	4.1	0.04	10.9
		T3E2	*wPt3247-wPt1695*	0	4.2	0.05	9.6
		T3AV	*wPt3247-wPt1695*	1	5.9	0.04	14.4
	QTsc-7A	T3AV	*swes134b-ubc822b*	22	3.6	0.04	13.2
RSUE	*QRue-1B*	T2E1	*wPt0944-wPt5363*	0	3.2	-2.03	10.0
	QRsue-1D	T3E1	*wPt665480-wPt666067*	0	6.5	-1.37	16.9
	QRsue-2A	T1E2	*swes217b-gwm71b*	1	3.8	-2.54	10.1
	QRsue.1-2B	T1E1	*wPt0100-wPt6627*	3	6.0	-2.68	20.1
	QRsue.2-2B	T3E2	*srap1a-gwm120*	4	3.1	-2.30	10.4
	QRsue.1-3A	T3E1	*wPt0836-wPt4725*	0	3.5	1.71	9.2
	QRsue.2-3A	T2E1	*barc1044-wPt664250*	2	4.1	4.91	16.2
	QRsue-3B	T1E2	*wPt0751-wPt7614*	2	3.9	2.48	11.7
	QRsue-4A	T3AV	*wPt4620-wPt5172*	0	3.1	-1.01	8.3
	QRsue-5D	T3E2	*barc49a-wPt5505*	1	3.1	2.40	9.4
	QRsue.1-6A	T2E2	*swes119b-wmc163*	0	4.6	-3.25	14.4
	QRsue.2-6A	T3AV	*wPt671561-wPt664603*	0	5.2	1.60	14.9
	QRsue.3-6A	T1E2	*wPt4229-wPt731002*	2	8.1	3.76	26.1
		T1AV	*wPt7204-swes1062*	0	3.4	1.47	9.3
		T3E2	*wPt668031-wPt4229*	7	4.8	2.73	14.4
	QRsue-7B	T3E1	*wPt4025-wPt664593*	0	3.1	1.67	8.2
SSUE	*QSsue-1B*	T2E1	*wmc314-ubc834b*	8	5.4	7.45	15.6
		T2AV	*ubc834b-ubc880d*	0	3.1	4.07	8.1
	QSsue-1D	T1E1	*wmc432b-wPt665480*	9	4.4	-6.25	15.7
		T3E1	*wPt665480-wPt666067*	0	3.3	-5.30	8.9
		T1E2	*srap29b-GlluD1*	15	3.4	6.52	10.0
	QSsue-2A	T1E2	*swes217b-gwm71b*	4	4.5	-11.45	14.4
	QSsue-2B	T2E2	*wPt5374-wmc154a*	12	4.1	6.80	9.8
		T3E2	*wmc154b-wPt7970*	20	5.7	9.38	29.2
		T3AV	*wmc154b-wPt7970*	23	3.8	5.45	16.8
	QSsue-3A	T2AV	*wPt4692-ubc859e*	22	3.1	7.99	37.1
	QSsue-3B	T2E1	*wPt2416-issr25a*	0	4.6	-5.91	12.4
	QSsue-4A	T3E2	*wPt4620-wPt5172*	0	3.2	-4.90	7.6
	QSsue-4B	T3E2	*wPt7569-wPt3991*	0	3.5	-5.23	8.5
	QSsue-6A	T1AV	*wPt731524-wPt7204*	8	4.2	4.86	13.6
		T2E2	*wPt7204-swes1062*	0	3.2	5.34	7.2
		T2AV	*wPt7204-swes1062*	0	4.4	4.43	11.8
	QSsue-6B	T2E2	*wPt4924-wPt3060*	0	3.3	-5.28	7.7
	QSsue-7A	T3AV	*wPt4637-barc121*	0	6.1	-5.10	15.7
	QSsue-7B	T1E2	*wPt730651-wPt2305*	0	3.1	7.65	8.6
TSUE	*QTsue.1-1A*	T2E2	*wPt6654-wPt5316*	3	3.0	10.32	8.5
	QTsue.2-1A	T2AV	*wPt2847-wPt1973*	0	3.7	-7.76	10.7
	QTsue-1B	T2E1	*ubc834b-ubc880d*	2	3.8	7.14	12.1
	QTsue-1D	T1E1	*wmc432b-wPt665480*	8	4.8	-7.82	17.2

Table S5 Continued

Trait[a]	QTL	Enviroment	Marker interval	Site[b] (cM)	LOD	Additive Effect	R^2(%)
		T3E1	*wPt665480-wPt666067*	1	6.5	-8.13	18.8
	QTsue-2A	T1E2	*swes217b-gwm71b*	0	6.3	-16.77	16.6
	QTsue-3A	T1E1	*wPt666438-wPt4692*	0	3.2	5.62	8.9
		T2AV	*wPt666438-wPt4692*	17	5.1	6.25	16.3
	QTsue.1-3B	T3E2	*wPt1940-wmc418*	1	3.6	-6.66	10.0
	QTsue.2-3B	T1E2	*wPt0751-wPt7614*	0	3.4	8.27	8.9
		T2E1	*wPt2416-issr25a*	3	3.3	-6.30	11.0
	QTsue-4B	T3AV	*wPt7569-wPt3991*	12	3.5	-4.32	9.6
	QTsue-5D	T2E2	*barc49b-barc49a*	2	3.9	9.31	10.7
	QTsue.1-6A	T3E1	*wPt7475-wPt9075*	11	3.9	6.25	11.0
	QTsue.2-6A	T1E2	*wPt7204-swes1062*	0	4.4	9.54	12.4
		T1AV	*wPt7204-swes1062*	0	3.7	5.85	10.9
	QTsue-7D	T2AV	*wPt4555-gdm67*	2	3.5	-8.84	13.3

Table S6 Locations of QTL clusters of S-related traits in this paper and the other traits in previous studies.

Chromosome	QTL clusters	Markers	QTLs in this study				QTLs detected in previous study					
							Related traits					References
1A	C1	*wPt731490-wPt669484*	*QRsc-1A*	*QSh.1-1A*	*QSsc.1-1A*	*QTsc.1-1A*	Htkc	Hskc	Hrkc			Kong et al.2013
1D	C3	*wPt7946-GlluD1*	*QRdw-1D*	*QRsc-1D*	*QSdw-1D*	*QSh-1D*	Tkue	Skue	Rkue	Tpue	Spue	
			QSsue-1D	*QRSue-1D*	*QTsue-1D*	*QTdw-1D*	Rpue	Tnue	Snue	Tkc	Skc	
			QRn-1D	*QTsc-1D*	*QSsc-1D*		Rkc	Tpc	Spc	Tnc	Snc	
							Tdw	Sdw	Rdw	Sh		Guo et al.2012
							Hrkue	Pgkue	Hskc			Kong et al.2013
							Tdw	Sdw				Sun et al.2013
2B	C5	*wPt5374-wPt7970*	*QSdw-2B*	*QSsc-2B*	*QSsue-2B*	*QTdw-2B*	Rkue	Tnue	Snue			Guo et al.2012
			QTsc-2B	*QRn.1-2B*	*QSh.1-2B*		Htkc	Hskc				Kong et al.2013
							Sdw	Tdw				Sun et al.2013
3B	C6	*swes862-ubc853c*	*QRsr.1-3B*	*QSh.1-3B*	*QRsc.1-3B*	*QSsc-3B*	Pup	Sdw				Su et al.2009
			QTsc-3B									
4B	C9	*swes24c-wPt5334*	*QSh-4B*	*QSdw-4B*	*QSsue-4B*	*QTdw-4B*	Tkue	Skue	Tpue	Spue	Tnue	
			QTsc-4B	*QTsue-4B*	*QRn-4B*		Tkc	Skc	Rkc	Rpc	Tnc	
							Sdw	Snue	Rnc			Guo et al.2012
							Tdw	Sdw				Sun et al.2013
7A	C11	*wPt4637-barc121*	*QSsue-7A*	*QSsc.2-7A*	*QTdw-7A*	*QSdw-7A*	Sdw	Tdw				Sun et al.2013
			QRn.2-7A									

Note: Hrkc, Root K content per plant in hydroponic culture trial; Hskc, Shoot K content per plant in hydroponic culture trial; Htkc, Total K content per plant in hydroponic culture trial; Tkue, Total K utilization efficiency; Skue, Shoot K utilization efficiency; Rkue, Root K utilization efficiency; Tpue, Total P utilization efficiency; Spue, Shoot P utilization efficiency; Rpue, Root P utilization efficiency; Tnue, Total N utilization efficiency; Snue, Shoot N utilization efficiency; Tkc, Total K content per plant; Skc, Shoot K content per plant; Rkc, Root K content per plant; Tpc, Total P content per plant; Spc, Shoot P content per plant; Tnc, Total N content per plant; Snc, Shoot N content per plant; Tdw, Total dry weight per plant; Sdw, Shoot dry weight per plant; Rdw, Root dry weight per plant;

Hrkue, Root K utilization efficiency in hydroponic culture trial; Pgkue, Grain K utilization efficiency in Pot trial; Pup, P accumulated in the shoot per plant; Rpc, Root P content per plant; Rnc, Root N content per plant.
